# Transcriptomic profiling of intermediate cell carcinoma of the liver

**DOI:** 10.1097/HC9.0000000000000505

**Published:** 2024-08-05

**Authors:** Byungchan Jang, So Mee Kwon, Jang Hyun Kim, Jung Mo Kim, Taek Chung, Jeong Eun Yoo, Haeryoung Kim, Julien Calderaro, Hyun Goo Woo, Young Nyun Park

**Affiliations:** 1 Department of Physiology, Ajou University School of Medicine, Suwon, Republic of Korea; 2 Department of Biomedical Science, Graduate School, Ajou University, Suwon, Republic of Korea; 3 Ajou Translational Omics Center (ATOC), Research Institute for Innovative Medicine, Ajou University Medical Center, Suwon, Republic of Korea; 4 Department of Pathology, Graduate School of Medical Science, Brain Korea 21 Project, Yonsei University College of Medicine, Seoul, Republic of Korea; 5 Severance Biomedical Science Institute, Yonsei University College of Medicine, Seoul, Republic of Korea; 6 Department of Pathology, Seoul National University College of Medicine, Seoul, Republic of Korea; 7 Department of Pathology, Assistance Publique Hôpitaux de Paris, Groupe Hospitalier Henri Mondor, Créteil, France

## Abstract

**Background:**

Intermediate cell carcinoma (Int-CA) is a rare and enigmatic primary liver cancer characterized by uniform tumor cells exhibiting mixed features of both HCC and intrahepatic cholangiocarcinoma. Despite the unique pathological features of int-CA, its molecular characteristics remain unclear yet.

**Methods:**

RNA sequencing and whole genome sequencing profiling were performed on int-CA tumors and compared with those of HCC and intrahepatic cholangiocarcinoma.

**Results:**

Int-CAs unveiled a distinct and intermediate transcriptomic feature that is strikingly different from both HCC and intrahepatic cholangiocarcinoma. The marked abundance of splicing events leading to intron retention emerged as a signature feature of int-CA, along with a prominent expression of Notch signaling. Further exploration revealed that *METTL16* was suppressed within int-CA, showing a DNA copy number–dependent transcriptional deregulation. Notably, experimental investigations confirmed that *METTL16* suppression facilitated invasive tumor characteristics through the activation of the Notch signaling cascade.

**Conclusions:**

Our results provide a molecular landscape of int-CA featured by *METTL16* suppression and frequent intron retention events, which may play pivotal roles in the acquisition of the aggressive phenotype of Int-CA.

## INTRODUCTION

The majority of primary liver carcinoma is comprised of HCC and intrahepatic cholangiocarcinoma (iCCA). However, this dichotomous classification is challenging because of the existence of intermediate tumors between them. Classical HCCs and iCCAs exhibit hepatocytic and cholangiocytic differentiations, respectively. Whereas there exists a combined hepatocellular-cholangiocarcinoma and CCA (cHCC-CCA), which is a primary liver cancer characterized by the unequivocal presence of both HCC and iCCA within the same tumor.^1^^,^^2^

Intermediate cell carcinoma (Int-CA) is a rare and unique primary liver carcinoma characterized by its monotonous morphological features, comprising intermediate tumor cells that exhibit characteristics lying between hepatocytes and cholangiocytes at the cellular level. Importantly, there are no areas showing histopathological features HCC or iCCA in int-CA.^3^^,^^4^ The tumor cells are small and uniform with scanty cytoplasm and hyperchromatic nuclei, and they are arranged in cords, strands, or trabeculae in a broad desmoplastic stroma. There is no mucin production.^5^ Both hepatocyte markers (eg, HepPar1, arginase-1, and α-fetoprotein) and cholangiocyte markers (eg, cytokeratin 19 and carcinoembryonic antigen) are expressed in the tumor cells of int-CA. According to the World Health Organization’s (WHO) Classification of Tumors of the Digestive System 5th ed, it remains unclear whether int-CA is a distinct subtype of cHCC-CCA or a histological pattern of cHCC-CCA, as its molecular characteristics have not yet been fully elucidated.^6^^,^^7^

Previously, molecular profiling and pathological studies have shown that there is a spectrum between HCC and iCCA and that those expressing progenitor cell–like traits show worse prognostic outcomes.^8^^–^^10^ The cHCC-CCAs also have been suggested to have a worse prognosis and express several hepatic progenitor cell markers, implying the bipotential liver progenitor cell origin.^1^^,^^11^ cHCC-CCAs were reported to be classified into 2 subtypes, combined and mixed, based on their growth pattern. Mixed-type cHCC-CCAs were defined as tumors with intimately mixed components of HCC and iCCA in the same tumor without clear boundaries.^7^ It is clearly different from int-CA, which is composed of the morphologically homogeneous tumor cells intermediates between hepatocytes and cholangiocytes at the cellular level. With this concern, molecular profiling studies of int-CA have not been performed thoroughly. In this study, we performed and analyzed RNA sequencing (RNA-seq) and whole genome sequencing (WGS) profiling of int-CA. Compared with the molecular features of HCCs and iCCAs, we demonstrated that int-CA exhibits a unique molecular feature of DNA copy number loss and concomitant transcriptional suppression of *METTL16*, which contributes to *Notch* activation. It may facilitate stem cell–like traits and invasive characteristics of int-CA. Based on these unique and distinct molecular-pathological characteristics, int-CA is suggested to be a distinct subtype of cHCC-CCA rather than a spectrum of histological patterns.

## METHODS

### Patients, tissue specimens, and pathological evaluation

The patients who had been diagnosed with int-CA, HCC, or iCCA and had undergone curative hepatic resection between 2012 and 2022 at Severance Hospital, Yonsei University Health System (n = 16), Seoul National University Hospital (n = 1) in Korea, and at Assistance Publique Hôpitaux in France (n = 1) were included. A total of 25 formalin-fixed, paraffin-embedded (FFPE) tissue specimens were used in this study, including int-CA (n = 8), HCC (n = 5), iCCA (n = 5), and their pair-matched nontumor liver tissues (n = 7). Cases of HCC and iCCA were randomly selected from patients with T1 and T2 stages. No patients were administered any preoperative treatment.

The pathological diagnosis of int-CA, HCC, and iCCA was performed according to the WHO Classification of Tumors of the Digestive System 5th ed.^7^^,^^12^^,^^13^ Immunohistochemical stain for hepatocytic (HepPar1), cholangiocytic markers (cytokeratin 19), nestin, and Notch1 was performed in int-CAs. The Institutional Review Board of Severance Hospital approved this study (no. 4-2022-0190) and waived the requirement for informed consent.

### RNA-Seq profiling

Total RNA from int-CA, HCC, iCCA, and nontumor liver FFPE tissues were extracted using the RNeasy Mini Kit (Qiagen). The mRNA sequencing library was prepared using the Truseq RNA Exome Kit (Illumina) according to the manufacturer’s instructions. RNA-Seq data were obtained using an Illumina NovaSeq for 151 bp paired-end reads with coverage greater than 48 million reads per sample. Sequence reads were aligned to the genome reference consortium human build (GRCh38) with the STAR method (version; v2.7.3.a).^14^ RNA abundance was estimated using Tophat2 and Cufflinks.^15^^,^^16^

### Public data analyses

Public data of liver cancer samples with transcriptome profiles, including GSE32879, GSE35306, and GSE179443, were obtained from the GEO database (https://www.ncbi.nlm.nih.gov/geo/). The DNA copy number profiles and their matched transcriptome profiles of HCC and iCCA were obtained from the Cancer Genome Atlas (TCGA, https://portal.gdc.cancer.gov/) and GSE76311. Normal and recurrent tumor samples were excluded from the analysis. Finally, we pooled our data with the publicly available data of primary HCCs (n = 513), iCCAs (n = 203), and cHCC-CCAs (n = 34), which were obtained from 6 independent data sets of TCGA-LIHC (n = 368) TCGA-CHOL (n = 30), GSE76311 (n = 152), GSE35306 (n = 30), GSE32879 (n = 23), and GSE179443 (n = 137). Batch effects across the studies were corrected by using the Combat method implemented in the R package. The stromal score and tumor purity of each sample were evaluated using the ESTIMATE method in the R package.^17^

### WGS and DNA copy number analysis

Total DNA from int-CA and nontumor FFPE tissues were extracted using the QIAamp FFPE Tissue Kit (Qiagen). The sequencing library for WGS was constructed using TruSeq Nano DNA library prep Kit (Illumina) according to the manufacturer’s instructions. WGS profiling was performed using an Illumina NovaSeq for 151 bp paired-end reads with coverage greater than 825 million reads per sample. Briefly, the sequence reads were aligned to the genome reference consortium human build (GRCh38) using BWA.^18^ Picard was used to remove PCR duplicates and coordinate sorting the reads. DNA copy number aberrations (CNAs) were estimated using the CNVkit^19^ implemented in the R package. Chromosomal cytobands with differentially altered DNA copy numbers between the tumor types were identified using the criteria of the fold difference of CNAs >0.2 and permutated *t* test *p* < 0.05.

### Estimation of splicing events

Alternative splicing events were detected and estimated by SplAdder.^20^ Intron retention (IR) was estimated by iREAD.^21^ Sequence reads with introns with total fragments <10 or without values for junction reads, fragments per kilobase of transcript per million, or entropy scores were filtered out. The expression levels of the IR transcripts were estimated by the STAR method (https://github.com/alexdobin/STAR/releases).

#### Knockdown and overexpression of METTL16

Knockdown of *METTL16* was performed by transfecting si*METTL16* (10 nM) for 12 hours. The overexpression vector for *METTL16* (RC208648) was constructed using pcDNA3.1 (0.1 μg) and transfected for 24 hours. Lipofectamine RNAiMAX was used as the transfection reagent. Negative Control small-interfering RNA is used as control.

### Cell culture and molecular experiments

Cell culture and molecular experiments were performed, including western blotting, real-time qPCR, immunohistochemistry, and invasion and proliferation assays. The details of the experiments were described in Supplemental Materials and Methods, http://links.lww.com/HC9/B10.

### Statistical analysis

For transcriptomic analysis, the correlation coefficient and *p* value were calculated based on Pearson’s product-moment correlation, and all statistical tests were performed using R software (version 4.2.2; Vienna, Austria).

## RESULTS

### Clinicopathological features of int-CAs

**FIGURE 1:**
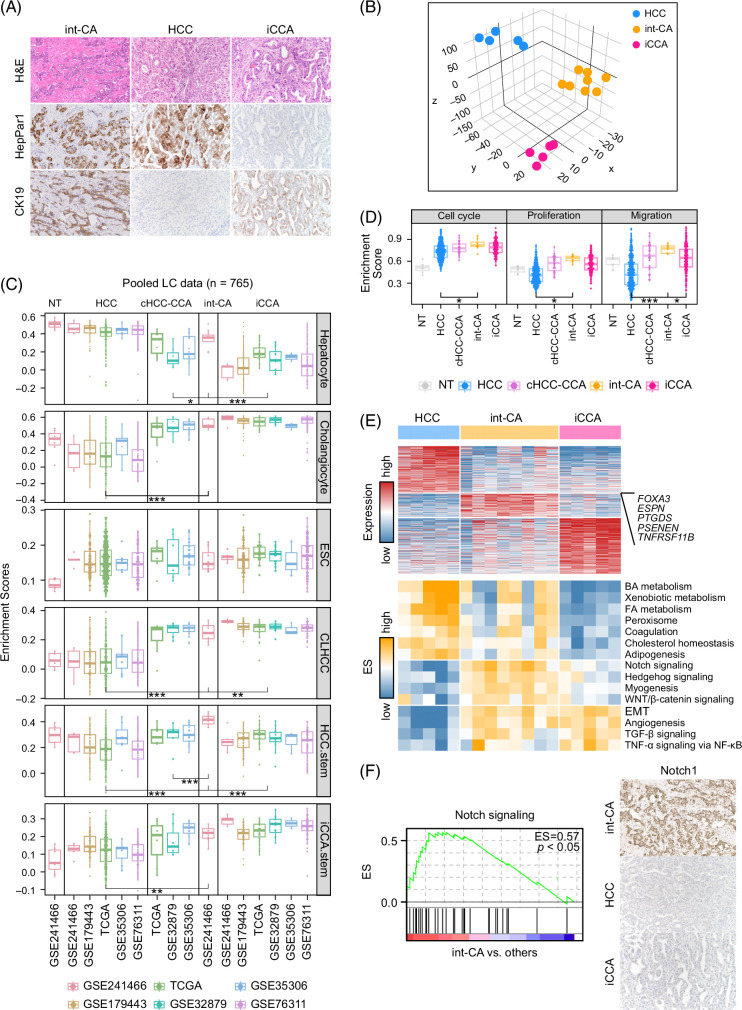
Comparison of the transcriptome profiles across HCC, iCCA, and int-CA. (A) Histopathological and immunohistochemical stain features of int-CA are shown. The tumor cells show coexpression of hepatocytic (hepPar1) and cholangiocytic (cytokeratin 19) markers. (B) Principal component analysis using the variable genes (median absolute deviation >0.5, n = 9847) shows the distinct distribution of int-CA compared to HCC and iCCA. (C) Box plots show the differential expression of the genes related to cellular origin across the tissue types such as hepatocyte, cholangiocyte, ESCs, and the stem cell–like features of liver cancer cells such as CLHCC, HCC.stem, and iCCA.stem. (D) Box plots show the enrichment scores of the genes for cell cycle, proliferation, and migration across the tissue types. (E) Heatmaps show the DEGs for each tissue type of HCC (n = 251), int-CA (n = 132), and iCCA (n = 302) (top), and the enrichment scores calculated by ssGSEA of the hallmark gene sets (n = 50, MSigDB, https://www.gsea-msigdb.org) across the tissue types (bottom). (F) The enrichment score of “Notch signaling” in int-CA compared to other tumor types is shown (left). Immunohistochemical stain for NOTCH1 shows a positive expression in int-CA (right). Abbreviations: CLHCC, cholangiocarcinoma-like hepatocellular carcinoma; DEG, differentially expressed gene; ESC, embryonic stem cell; iCCA, intrahepatic cholangiocarcinoma; int-CA, intermediate cell carcinoma; ssGSEA, single sample gene set enrichment analysis.

We selected 8 patients with int-CAs. The mean (range) age was 54.1 (46–68) years, and the male-to-female ratio was 7:1. The mean (range) tumor size was 3.89 (1.5–8.7) cm. Histologically, int-CAs exhibited a monomorphic feature throughout the entire tumor, and they were composed of intermediate cells between hepatocytes and cholangiocytes at the cellular level. The tumor cells were small and oval-shaped with hyperchromatic nuclei and scant cytoplasm. They were arranged in trabeculae, solid nests, or strands in the abundant fibrous tumor stroma. The tumor cells of int-CAs showed coexpression of hepatocytic (HepPar1, arginase-1, or α-fetoprotein) and biliary markers (cytokeratin 19 or carcinoembryonic antigen), and at least 1 of the hepatocyte markers and 1 of the biliary markers were detected by immunohistochemical stain (Figure 1A). The tumor border showed infiltrative growth without a tumor capsule, and vascular invasion was noted in 5 cases (62.5%). The areas of HCC or CCA were not found, and mucin production was not present. The histopathology of the background liver showed chronic B viral hepatitis/cirrhosis in all cases. Two patients showed local recurrence at 976 days and 1182 days after resection, respectively, and all patients were alive except a patient with concurrently progressive HCC during the follow-up period (mean, 2231.3 d; range, 593–2645 d). The overall clinicopathological features of the patients are summarized in Supplemental Table S1, http://links.lww.com/HC9/B10, and Supplemental Table S2, http://links.lww.com/HC9/B10.

### Transcriptomic comparison of int-CA, HCC, and iCCA

To compare the molecular characteristics across liver cancer types, we performed transcriptomic profiling of int-CA (n = 8), HCC (n = 5), and iCCAs (n = 5) using FFPE tissue samples (GSE241466). Unsupervised cluster analysis and principal component analysis revealed that the transcriptome profiles of each tumor type were clustered together, revealing a distinct and intermediate molecular feature of int-CA between HCC and iCCA (Figure 1B and Supplemental Figure S1, http://links.lww.com/HC9/B10). This finding is in agreement with previous studies showing intermediate morphological characteristics of int-CA lying between those of HCC and iCCA.^3^ Next, to broaden the scope of our study and compare the results with those of other tumor types, we pooled our data with publicly available data on primary HCCs (n = 513), iCCAs (n = 203), and cHCC-CCAs (n = 34) derived from 6 independent data sets (for details see Methods). We compared the expression levels of marker genes across each tumor type in the study cohorts, demonstrating that our findings were not confounded by the study cohorts. We demonstrated an intermediate phenotype of int-CA, which expressed marker genes for both hepatocytes and cholangiocytes at an intermediate level between those observed in HCC and iCCA (Figure 1C, top and Supplemental Figures 2A, B, http://links.lww.com/HC9/B10). We also examined the expression of *NESTIN,* which has been addressed as a diagnostic and prognostic marker for cHCC-CCA.^22^^,^^23^ Int-CA showed higher expression of *NESTIN* compared to that of cHCC-CCA (Supplemental Figure 2C, left, http://links.lww.com/HC9/B10), and nestin protein was detected in all cases of int-CAs by immunohistochemical stain (Supplemental Figure 2C, right, http://links.lww.com/HC9/B10), suggesting that nestin is a marker of int-CA. In addition, we compared the expression levels of the progenitor cell–related genes across tumor types (Supplemental Table S3, http://links.lww.com/HC9/B10). The expression levels of embryonic stem cell–related genes were not significantly different among tumor types (Figure 1C, middle). However, int-CA demonstrated higher expression of cholangiocarcinoma-like HCC-related genes than that of HCC-related genes, similar to cHCC-CCA.^8^ The int-CAs also highly expressed cancer stem cell features of HCC (HCC.stem) and iCCA (iCCA.stem) compared to those of cHCC-CCA (Figure 1C, bottom). Our results suggest that int-CA is a more primitive subtype than cHCC-CCA, expressing bipotential progenitor-like traits but not expressing pluripotent stem cell (ie, embryonic stem cell) traits. Moreover, compared to other cancer types, int-CA showed higher expression of cell cycle-related, proliferation-related, and migration-related genes (Figure 1D), supporting invasive characteristics of int-CA.

Next, to delineate the functional characteristics of each tumor type, we determined the differentially expressed genes (DEGs) for each tumor type of HCC (DEG_HCC_, n = 251), int-CA (DEG_int-CA_, n = 132), and iCCA (DEG_iCCA_, n = 302), respectively (fold difference >1, permuted *t* test, *p* < 0.01; Figure 1E, top and Supplemental Table S4, http://links.lww.com/HC9/B10). Gene set enrichment analysis also revealed the differentially enriched signaling pathways across the tumor types (n = 18, fold difference >0.1, permuted *t* test, *p* < 0.05; Figure 1E, bottom). The int-CAs expressed the genes related to the Notch, hedgehog, myogenesis, and WNT/β-catenin signaling, whereas HCCs expressed metabolism-related genes and iCCA expressed epithelial-mesenchymal transition-related genes, respectively. Noticeably, int-CA, compared to HCC or iCCA, showed the most prominent expression of Notch signaling pathway in the gene set enrichment analysis between int-CA and other tumor types (enrichment score = 0.57, *p* < 0.05; Figure 1F, left). We further validated this finding by performing an immunohistochemical analysis of Notch1 expression (Figure 1F, right), which suggested that Notch activation plays an important role in the progression of int-CA.

### Comparison of the DNA copy numbers across liver cancer subtypes

**FIGURE 2:**
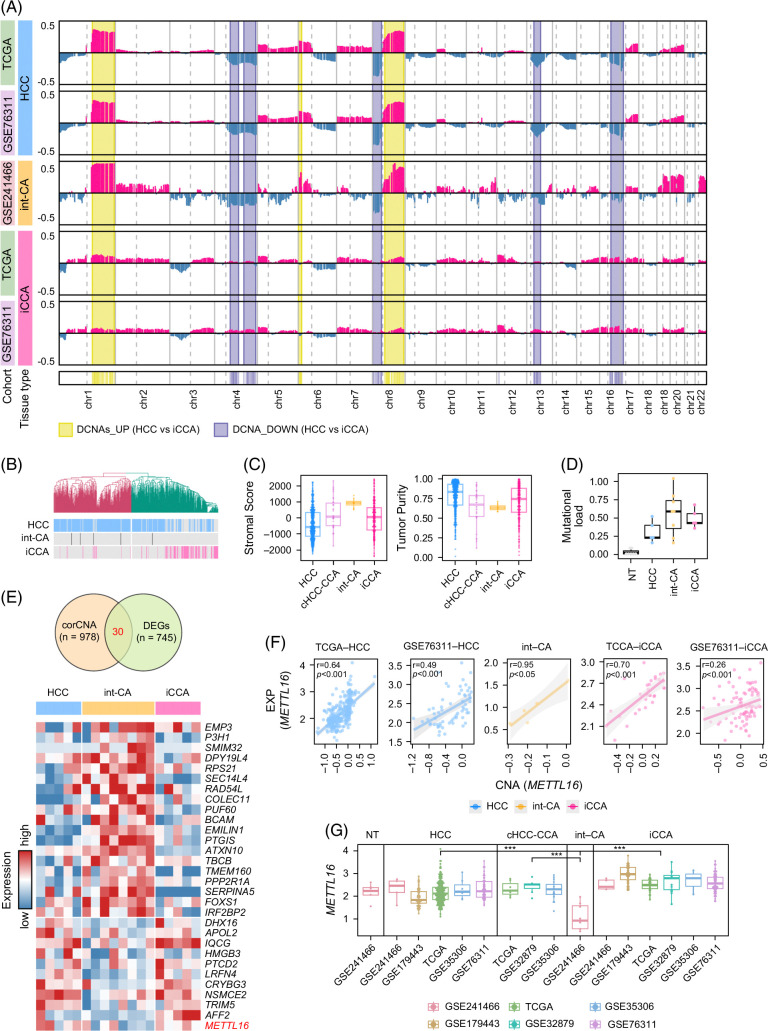
DNA copy number–dependent transcriptional deregulation of *METTL16.* (A) The CNA values across the tumor types are shown in various data sets of TCGA-HCC, GSE76311-HCC, int-CA, TCGA-iCCA, and GSE76311-iCCA (top). The chromosomal regions with differentially altered CNAs with gain or loss are indicated (bottom, colored boxes). (B) Unsupervised cluster analysis of the CNA profiles shows the similarity of the CNAs across the tumor types. (C) Stromal score (left) and tumor purity (right) are shown across the tumor types. (D) Mutational load (mutations/Mb) across the tissue types is shown. (E) A Venn diagram shows the CNA-dependent genes identified from the overlapped genes among the correlated CNAs and the DEGs for int-CA (top). A heatmap shows the expression of CNA-dependent genes in int-CA (n = 30, bottom). (F) Correlations between the expression levels and the CNA levels of *METTL16* are shown across the tumor types in the data sets of int-CA, TCGA-HCC, GSE76311-HCC, TCGA-iCCA, and GSE76311-iCCA, respectively. (G) A boxplot shows *METTL16* expression across the tumor types in the various data sets (*p* < 0.05). Abbreviations: CNA, copy number aberration; DEG, differentially expressed gene; iCCA, intrahepatic cholangiocarcinoma; int-CA, intermediate cell carcinoma; TCGA, The Cancer Genome Atlas.

Next, we evaluated the DNA copy number aberration (CNAs) of int-CA by performing a WGS (n = 5) (for details, see Methods). By pooling our data with the publicly available data sets of TCGA-HCC, TCGA-iCCA, GSE76311-HCC, and GSE76311-iCCA, we identified differentially altered CNAs between HCC and iCCA. HCCs had 58 differentially altered CNA regions at 1q21.2-44, 6p25.3-22.3, and 8q12.1-24.3, while iCCA had 66 differentially altered CNA regions at 4q13.3-25, 4q28.3, 4q31.21-35.1, 8p23.3-11.21, 12p13.2, 13q13.1-21.31, 16q11.2-24.3, and 17p13.3-12 (fold difference >0.2, permuted *t* test, *p* < 0.05; Figure 2A and Supplemental Table S5, http://links.lww.com/HC9/B10). Interestingly, int-CA revealed similar aberrations with the CNAs for HCC, showing higher amplification of 1q and 8q, along with more frequent deletion of 4p, 8p, 13q, and 17p. Indeed, unsupervised clustering of the CNAs revealed that most of the int-CA samples (4 out of 5) were clustered together with HCCs, indicating that int-CA is genomically more similar to HCC than iCCA (Figure 2B). This finding may contradict the intermediate features of the transcriptome profile of int-CA. The observation of fewer CNAs in iCCAs might be attributed to the lower purity of iCCA compared to that of HCC. To determine the effects of tumor purity on CNA estimation, we calculated tumor purity across tumor types (see Methods). Both cHCC-CCAs and int-CAs, compared to HCCs or iCCAs, showed lower tumor purity with rich stromal scores (Figure 2C). This result indicates that similar CNAs in int-CA and HCC may not be related to tumor purity. In addition, int-CA revealed a higher mutation rate (average 0.365 mutations/MB) compared to the other tumor types (HCC, 0.164 mutations/MB; CCA, 0.284 mutations/MB; Figure 2D), which was not related to the different tumor purity according to tumor types. Taken together, we suggest that int-CA exhibited unique genomic features of recurrent CNAs and higher mutational burden.

As the DNA copy number–dependent transcriptional dysregulation is thought to play a potential driver role in cancer progression, we identified the CNA-correlated genes in int-CA (n = 978, Pearson correlation coefficient *r* > 0.5, *p* < 0.05). Among them, we further determined that the CNA genes overlapped with the DEGs as the CNA-dependent DEGs (n = 30), which may represent the potential regulatory genes driving distinct transcriptional alteration int-CA (Figure 2E, top). Remarkably, we found that *METTL16* at 17p13.3 had prominent DNA copy loss with concomitant transcriptional suppression (*r* = 0.95, *p* < 0.05, Pearson correlation test; Figure 2E, bottom). The DNA copy–correlated expression of *METTL16* was validated in independent data sets of HCC (TCGA-LIHC and GSE76311-HCC) and iCCA (TCGA-CHOL and GSE76311-CCA) (*p* < 0.001), respectively (Figure 2F). We also confirmed that *METTL16* was significantly suppressed in int-CA compared to that in HCC, iCCA, cHCC-CCA, or NT (permutated *t* test, *p* < 1×10^−15^; Figure 2G). Thus, we suggest that DNA copy–dependent transcriptional suppression of *METTL16* is a unique feature of int-CA, playing an important role in int-CA progression.

### Splicing variant of IR is frequent in int-CA

**FIGURE 3:**
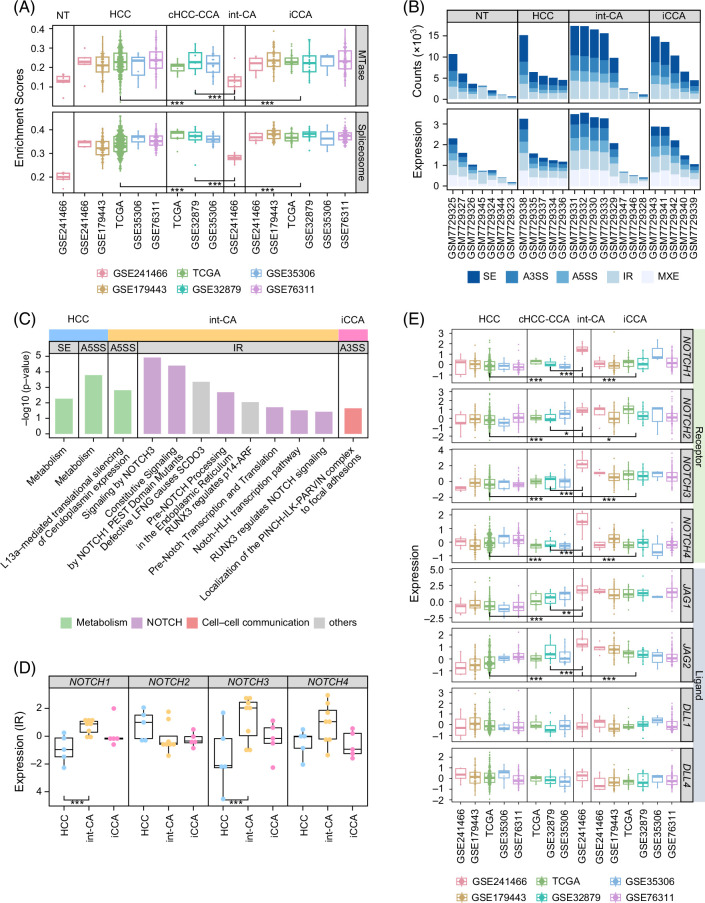
int-CA had frequent intron retention. (A) Box plots show the RNA methyltransferase activity and spliceosome activity across the tumor types. (B) Bar plots show the counts of the alternative splicing events and the expression levels of the spliced transcripts across the tumor types. (C) Bar plots show the enrichment scores for the expression of the pathway genes for each splicing type across the tumor types. (D) Box plots show the expression levels of IR transcripts in Notch receptors across the tumor types. (E) Box plots show the expression levels of Notch receptors (ie, *NOTCH1*, *NOTCH2*, *NOTCH3*, and *NOTCH4*) and ligands (ie, *JAG1*, *JAG2*, *DLL1*, and *DLL4*) across tumor types. Abbreviations: int-CA, intermediate cell carcinoma; IR, intron retention.

Although the functions of *METTL16* are not yet fully understood, *METTL16* enhances RNA binding activity and RNA methyltransferase (MTase) activity, modulating the splicing of the retained intron by regulating *MAT2A* expression.^24^ In line with this finding, we observed that mRNA MTase-related genes from gene ontology (n = 20, GO:0008174, mRNA methyltransferase activity) were significantly suppressed in int-CA compared to HCC or iCCA (permuted *t* test, *p* < 0.001; Figure 3A, top). We also evaluated spliceosome activity across the tumor types by calculating the enriched expression of the previously reported splicing-regulatory genes (n = 274),^25^ revealing significant suppression of the splicing-regulatory genes in int-CA compared to those in HCC or iCCA (permuted *t* test, *p* < 0.001; Figure 3A, bottom). This result supports that the splicing process in int-CA is significantly dysregulated. In addition, we further evaluated the frequencies of the splicing events for each type, including skipped exons, alternative 3ʹ splicing sites, alternative 5ʹ’ splicing sites (A5SSs), mutually exclusive exons, and IR (see Methods). All splicing events were more frequent and highly expressed in int-CA than in HCC or iCCA (Figure 3B). Taken together, these results suggest that the altered splicing process by the suppression of *METLL16* plays an important role in the development and progression of int-CA.

Next, we evaluated whether the dysregulated splicing processes were functionally enriched. We found that HCC was enriched in the expression of skipped exon and A5SS events of the metabolism-related pathways (Figure 3C). Notably, int-CA showed frequent IR events in the Notch-related signaling, which consistently supports that Notch is highly expressed in int-CA. In addition, we estimated the abundance of IR transcripts of Notch receptors (see Methods). The int-CAs showed higher expression of IR transcripts of *NOTCH1* and *NOTCH3* compared to the other tumor types but did not have the IR transcripts of *NOTCH2* and *NOTCH4* (*p* < 0.05; Figure 3D). Furthermore, we compared the gene-level (not the IR) expression of Notch receptors (ie, *NOTCH1*, *NOTCH2*, *NOTCH3*, and *NOTCH4*) and ligands (ie, *JAG1* [jagged canonical notch ligand 1], *JAG2* [jagged canonical notch ligand 2], *DLL1* [delta like canonical notch ligand 1], and *DLL4* [delta like canonical notch ligand 4]) using the pooled data of liver cancer, demonstrating that int-CA prominently expressed Notch ligands and receptors compared to the other tumor types (Figure 3E). In particular, int-CA prominently expressed the Notch ligands of *JAG1* and *JAG2* but not *DLL1* and *DLL4.* This finding implies that JAG signaling rather than DLL plays an important role in the activation of the Notch pathway in int-CA. We also observed that int-CAs, compared to the other tumor types, had higher expression of Notch coactivators (ie, *MAML1*) and target genes (ie, *HES1*, *HEY1*, and *HYEL*) (Supplemental Figure S3, http://links.lww.com/HC9/B10). These results consistently indicate that the Notch pathway is significantly activated in int-CA compared to that in other tumor types.

### *METTL16* suppresses *NOTCH1* expression

**FIGURE 4:**
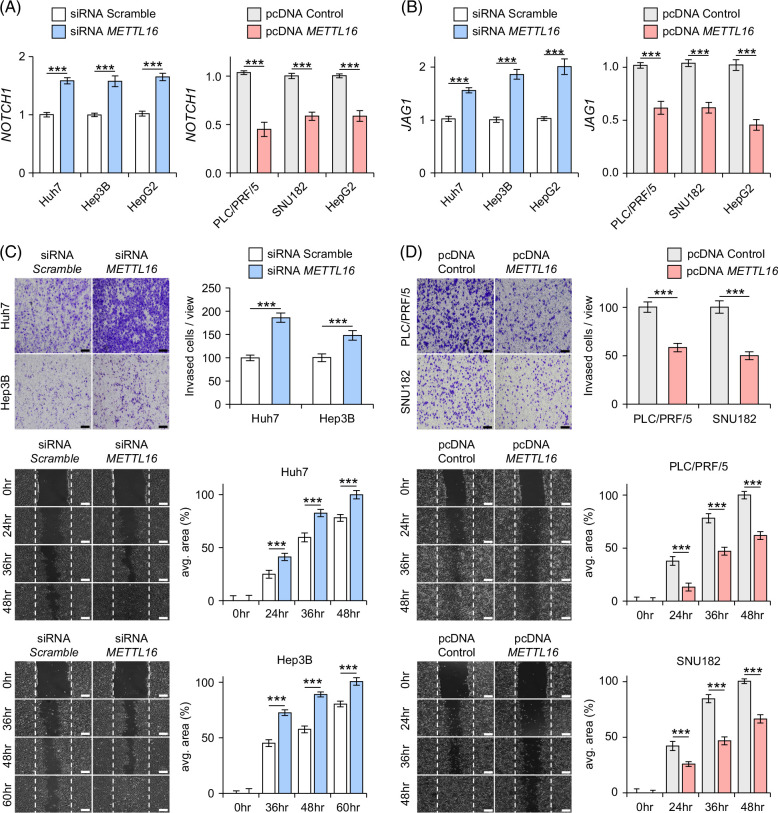
*METTL16* expression suppresses cancer cell invasion and migration. (A, B) Effects of siRNA-mediated *METTL16* knockdown (left) and overexpression (right) on the expression levels of *NOTCH1* (A) and *JAG1* (B). The data represent the mean ± SD of 6 independent experiments. (C, D) *METTL16* is knocked down by siRNA transfection in HuH7, Hep3B, and HepG2 cells (C) and overexpressed in PLC/PRF/5, SNU182, and HepG2 cells (D) invasion (top) and migration (bottom) are shown, respectively. ***p < 0.001. Abbreviation: siRNA, small-interfering RNA.

To evaluate the effects of *METTL16* on liver cancer cells (ie, Huh7, Hep3B, HepG2, PLC/PRF/5, and SNU182), we performed small-interfering RNA–mediated knockdown and overexpression experiments for *METTL16* using liver cancer cell lines (Supplemental Figure S4A, http://links.lww.com/HC9/B10). When *METTL16* expression was knocked down by transfecting small-interfering RNAs, *NOTCH1* expression was significantly induced in the different cell lines (Huh7, Hep3B, and HepG2; Figure 4A, left). In contrast, overexpression of *METTL16* in different cell lines (PLC/PRF/5, SNU182, and HepG2) suppressed *NOTCH1* expression (Figure 4A, right). We also examined the expression levels of Notch ligands. *JAG1* expression was significantly increased by knockdown of *METTL16,* whereas it decreased by the *METTL16* overexpression (Figure 4B). However, the expression levels of the other Notch receptors (*NOTCH3* and *NOTCH4*) and ligands (*JAG2*, *DLL1*, and *DLL4*) were not significantly altered by perturbation of *METTL16* (Supplemental Figures S4B–D, http://links.lww.com/HC9/B10). Together with the results from the experiments and RNA-Seq data of human tissues (Figure 3E), we suggest that *METTL16* expression suppresses Notch signaling, at least in part, through NOTCH1 and JAG1.

Next, we investigated whether *METTL16* expression affected the phenotype of liver cancer cells. We demonstrated that the knockdown of *METTL16* accelerated the invasion and migration of liver cancer cells (Figure 4C). Vice versa, overexpression of *METTL16* significantly suppressed the invasion and migration of liver cancer cells (Figure 4D). These results suggest that *METTL16* suppression contributes to the acquisition of invasive and migratory phenotypes. However, the proliferation of cancer cells was not affected by the perturbation of *METTL16* (Supplemental Figure S5, http://links.lww.com/HC9/B10). Kaplan-Meier’s survival analysis revealed no significant associations between *METTL16* expression and the prognostic outcomes of patients with HCC or iCCA in 2 independent data sets (Supplemental Figure S6, http://links.lww.com/HC9/B10).

**FIGURE 5:**
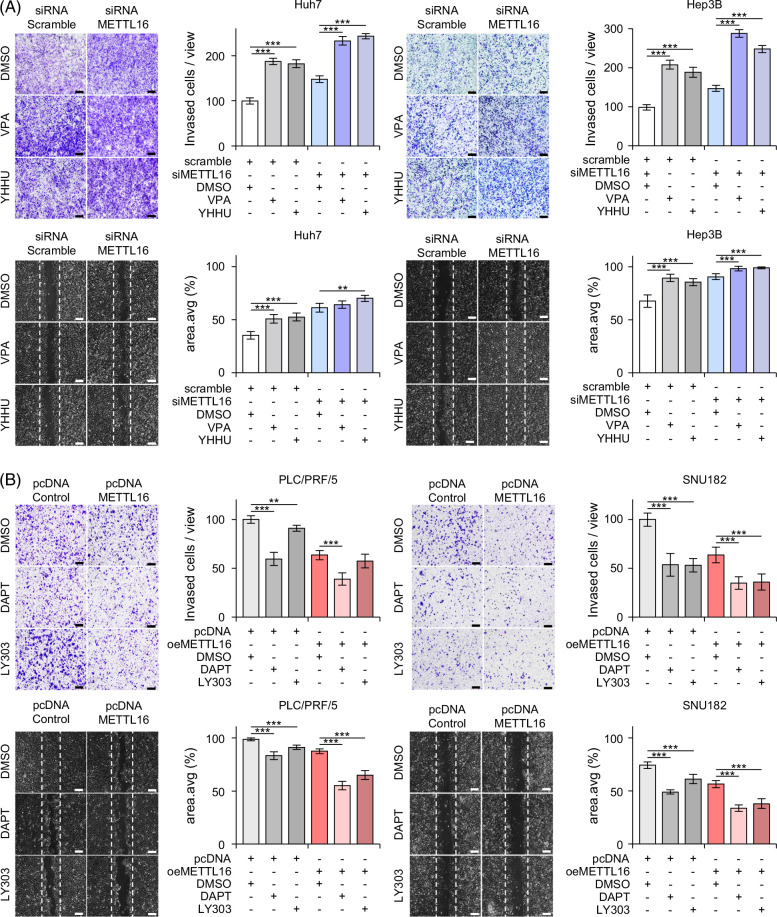
Notch signaling mediates the effect of *METTL16* on cancer cells. (A) Effects of Notch activators (valproic acid, VPA, 1 mM; YHHU3792, YHHU, 5 μM) on the invasion (top) and migration (bottom) of the siMETTL16-treated HuH7 (left) and Hep3B (right) cells are shown, respectively. (B) Effects of Notch inhibitors (DAPT, 10 μM; Crenigacestat, LY303, 2 nM) on the invasion (top) and migration (bottom) of the siMETTL16-treated PLC/PRF/5 (left) and SNU182 (right) cells are shown, respectively. Images are captured by light microscope (×5). Scale bars = 200 μm. **p < 0.01, and ***p < 0.001. Abbreviation: LY303, Crenigacestat.

In addition, as Notch activation was found to be a key feature of int-CA, we evaluated whether Notch signaling mediates the enhanced invasion and migration noted by *METTL16* suppression. Effects of *Notch* inhibitors (ie, DAPT [gamma secretase inhibitor] and LY303 [Crenigacestat]) and *Notch* activators (ie, valproic acid and YHHU [YHHU3792]) on *NOTCH1* expression (Supplemental Figure S7, http://links.lww.com/HC9/B10) were examined. Treatment with *Notch* activators augmented *METTL16* knockdown–mediated cancer cell invasion and migration (Figure 5A). Conversely, treatment with *Notch* inhibitors invalidated *METTL16* overexpression–mediated suppression of cancer cell invasion and migration (Figure 5B). Taken together, these results suggested that *METTL16* suppression in int-CA contributes to *Notch* activation, at least in part, by inducing *JAG1* and *NOTCH1* expression, resulting in the acquisition of invasive and migratory phenotypes.

## DISCUSSION

The classification of cHCC-CCA remains challenging and controversial owing to their high intratumoral heterogeneity. In this study, we collected int-CA samples using stringent diagnostic criteria that showed homogeneous populations of intermediate cells in the entire tumor. By performing molecular profiling of int-CA compared with those of HCC, iCCA, and cHCC-CCA, we successfully demonstrated the distinct molecular features of int-CA differing from other tumor types of the liver. Similar to cHCC-CCAs, int-CAs express stem cell–like traits, suggesting a bipotential liver progenitor cell origin.

In particular, int-CA had DNA copy–dependent suppression of *METTL16* transcription. Although the functions of *METTL16* have not been fully elucidated, several functions of *METTL16* have been identified. *METTL16* is an N6-methyladenosine RNA methyltransferase, which is thought to exert both methyltransferase activity–dependent and –independent functions in the regulation of gene expression.^24^^,^^26^^,^^27^ *METTL16* recruits splicing factors to specific exons, which can influence whether they are spliced out. It can also target specific mRNAs for N6-methyladenosine modification, which alters their stability and translation. Previous studies have shown that *METTL16* expression is associated with several diseases, including cancer. *METTL16* promotes tumor growth and metastasis by regulating the expression of genes involved in cell proliferation, survival, and migration.^28^ For example, *METTL16* expression promotes the progression of diverse cancer types, including lung,^29^ breast,^30^ stomach,^31^ and liver cancers.^32^ In contrast, opposite results have shown an association between *METTL16* expression and favorable prognostic outcomes in pancreatic^33^ and liver cancers.^34^ These discrepant results imply that the functions of *METTL16* are complex and depend on the context of tissue types.^35^^–^^37^ *METTL16* also regulates the transcriptional splicing of oncogenes or tumor suppressor genes. With this concern, we observed frequent IR splicing events in int-CA, which may facilitate *METTL16* suppression (Figure 2D). Furthermore, apart from the methyltransferase activity, multifaceted functions of *METTL16* have been addressed. A recent study has shown that METTL16 expression in pancreatic cancers confers synthetic lethality to PARP inhibition, revealing a novel function of *METTL16* in homologous recombination repair, which is important for maintaining genome stability.^38^ Further extended studies are required to delineate the underlying mechanisms involved in the role of *METTL16* in int-CA progression.

In this study, *METTL16* suppression in int-CA is considered to contribute to *Notch* activation, at least in part, by inducing *JAG1* and *NOTCH1* expression. Actually, Notch signaling has been reported to support liver cancer stemness,^39^ and accordingly, int-CA revealed upregulated expression of bipotential progenitor-like trait and *NESTIN*.^22^^,^^23^ Notch signaling is also reported to promote HSC differentiation into myofibroblasts and liver fibrosis.^40^ These features are well matched with the histopathological characteristics of int-CA, showing primitive tumor cell features and abundant fibrous tumor stroma. Accordingly, int-CA revealed a higher proportion of HSCs and rich stromal scores.

The prognosis of cHCC-CCA is known to be worse than that of HCC after resection^7^ and intermediate between that of HCC and iCCA.^41^ Data on the prognosis of int-CA remain limited due to the small series and number of patients. Roberechts et al^42^ reported an aggressive clinical course of int-CA, and Kim et al^3^ showed that the prognosis of int-CA was intermediate between that of HCC and iCCA. Akiba et al^43^ reported that the prognosis of classical cHCC-CCA was similar to that of int-CA. In this study, 2 cases of int-CAs showed local recurrence, and all cases except a case of concurrent HCC survived during 2231.3 days of the mean follow-up period. It may potentially be attributed to the comparatively smaller tumor sizes and lower tumor stages observed in the int-CAs of this study compared to those in the previous report.^3^ Interestingly, the in vitro experiments in this study demonstrated that *METTL16* suppression contributed to *Notch* activation, resulting in the acquisition of an aggressive phenotype of migration and invasion but not proliferation. This correlates well with the invasive histopathological characteristics of int-CAs, showing infiltrative growth of the tumor border without tumor capsule formation and frequent microvascular invasion. Survival analysis of patients with HCC and iCCA revealed no significant associations between *METTL16* expression and prognostic outcomes in the 2 independent data sets (TCGA-HCC, TCGA-iCCA, GSE179443-HCC, and GSAE179443-iCCA). Therefore, further studies of int-CA based on larger cohorts are needed.

In conclusion, molecular profiling of int-CA can demonstrate the unique and distinct features of int-CA compared to those of HCC or iCCA, suggesting that int-CA is a specific subtype of primary liver carcinoma rather than a spectrum of histological patterns. Our results provide new pathobiological insights into the development and progression of int-CA. Targeting *METTL16* may be a promising therapeutic strategy for tumors with dysregulated expression of *METTL16* like int-CA.

## Supplementary Material
