# Supplementary material for: Transcriptomic profiling of intermediate cell carcinoma of the liver
Source: Hepatol Commun. 2024 Aug 5;8(8):e0505. doi: 10.1097/HC9.0000000000000505 (PMC11299988; doi:10.1097/HC9.0000000000000505)
Supplement: Supplementary file 1 [file hc9-8-e0505-s001.docx]

**Supplementary Information for:**

# Transcriptomic profiling of intermediate cell carcinoma of the liver

Byungchan Jang^1,2,†^, So Mee Kwon^1,†^, Jang Hyun Kim^1,2^, Jung Mo Kim^3^, Taek Chung^4^, Jeong Eun Yoo^4,5^, Haeryoung Kim^6^, Julien Calderaro^7^, Hyun Goo Woo^1,2, 3*^, Young Nyun Park ^4,5*^

^1^Department of Physiology, Ajou University School of Medicine, Suwon, Republic of Korea

^2^Department of Biomedical Science, Graduate School, Ajou University, Suwon, Republic of Korea

^3^Ajou Translational Omics Center (ATOC), Research Institute for Innovative Medicine, Ajou University Medical Center, Suwon, Republic of Korea.

^4^Department of Pathology, Graduate School of Medical Science, Brain Korea 21 Project, Yonsei University College of Medicine, Seoul, Republic of Korea

^5^Severance Biomedical Science Institute, Yonsei University College of Medicine, Seoul, Korea

^6^Department of Pathology, Seoul National University College of Medicine, Seoul, Republic of Korea

^7^Department of Pathology, Assistance Publique Hôpitaux de Paris, Groupe Hospitalier Henri Mondor, Créteil, France

^†^ These authors contributed equally to this work.

# Supplementary Materials and Methods

### *Immunohistochemical stain*

Immunohistochemical (IHC) staining was performed using representative sections of formalin-fixed, paraffin-embedded tissues. IHC staining was performed using an automated staining system (Ventana Medical Systems, Inc. Tucson, AZ) according to the manufacturer's instructions. In detail, the slides were deparaffinized using EZ Prep (Ventana Medical Systems) at 75°C for 4 minutes. Cell conditioning was performed using CC1 solution (Ventana Medical Systems) at 100°C for 30 to 60 minutes. Antibodies were diluted, followed by treatment, and incubated at 37°C for 32 minutes. Signals were detected using the UltraView DAB IHC Detection Kit (Ventana Medical Systems). Counterstaining was performed using Hematoxylin I (Ventana Medical Systems) for 4 minutes at room temperature.

| Primary antibody | Source | Dilution |
| --- | --- | --- |
| α-fetoprotein (Rabbit pAb) | DAKO (Glostrup, Denmark) | 1:800 |
| HepPar1 (mouse mAb, clone OCH1E5) | DAKO (Glostrup, Denmark) | 1:50 |
| Arginase-1 (rabbit mAb, clone SP156) | Cell marque  (Darmastadt, Germany) | 1:100 |
| Cytokeratin19 (mouse mAb, clone RCK108) | DAKO (Glostrup, Denmark) | 1:100 |
| Carcinoembryonic antigen (mouse mAb, clone Il-7) | DAKO (Glostrup, Denmark) | 1:200 |
| Nestin (mouse mAb, clone 10C2) | Merck (Darmastadt, Germany) | 1:400 |
| Notch 1 (mouse mAb, clone OTI3E12) | ORIGENE (Rockville, MD , US) | 1:50 |

Abbreviations: mAb, monoclonal antibody; pAb, polyclonal antibody

### *Cell Culture*

The human HCC cell lines Hep3B and HepG2 purchased from the American Type Culture Collection (ATCC, Manassas, VA, USA), and PLC/PRF/5, SNU182, and Huh7 purchased from the Korea Cell Line Bank (Seoul, Korea), were mainly grown in MEM (Gibco, Carlsbad, MD, USA), DMEM (Gibco) and RPMI (Gibco), respectively, supplemented with 10% fetal bovine serum (Gibco), 100 U/ml penicillin and 100 μg/mL streptomycin at 37 ℃ in a humidified atmosphere with 5% CO_2_.

### *Reagents*

AccuTarget™ Negative Control siRNA (SN^-10^01, BIONEER, USA), ON-TARGET plus Human METTL16 siRNA (L-016359^-02^-0005, Dharmacon, USA), pcDNA3.1 plasmid (Addgene, Cambridge, MA, USA), METTL16 (RC208648, ORIGENE, USA). DAPT(2634^-10^mg, Tocris), YHHU3792(6599^-10^mg, Tocris), Crenigacestat (S71690-25mg, selleckchem), and Valproic acid (HY^-10^585-1g, Medchemexpress) were used.

### *Reverse transcription and Quantitative PCR*

Cells were harvested and total RNAs were isolated using a RNeasy Plus Micro Kit (No. 74034; Qiagen, Netherlands). Reverse transcription of the total RNA (1μg) was performed using TOPscript RT DryMIX (RT200, enzynomics, Korea). Quantitative real-time PCR (qRT-PCR) was performed using the IQ SYBR Green Supermix (Bio-Rad, USA). The primer sequences and conditions are as follows.

| Primer  (F, forward; R, reverse) | Sequence (5’→3’) | Annealing Temperature |
| --- | --- | --- |
| *METTL16* F | TGGAGCAACCTTGAATGGCTGG | 59°C |
| *METTL16* R | CCATCAGGAGTGTCTTCTGTGG |  |
| *NOTCH1* F | GGTGAACTGCTCTGAGGAGATC | 59°C |
| *NOTCH1* R | GGATTGCAGTCGTCCACGTTGA |  |
| *NOTCH3* F | TACTGGTAGCCACTGTGAGCAG | 57°C |
| *NOTCH3* R | CAGTTATCACCATTGTAGCCAGG |  |
| *NOTCH4* F | CCTCCTATTTCTGCCACTGC | 56°C |
| *NOTCH4* R | ACAGGGTTGGGACTGACAAG |  |
| *JAG1* F | TGCTACAACCGTGCCAGTGACT | 59°C |
| *JAG1* R | TCAGGTGTGTCGTTGGAAGCCA |  |
| *JAG2* F | GCTGCTACGACCTGGTCAATGA | 59°C |
| *JAG2* R | AGGTGTAGGCATCGCACTGGAA |  |
| *DLL1* F | TGCCTGGATGTGATGAGCAGCA | 59°C |
| *DLL1* R | ACAGCCTGGATAGCGGATACAC |  |
| *DLL4* F | CTGCGAGAAGAAAGTGGACAGG | 59°C |
| *DLL4* R | ACAGTCGCTGACGTGGAGTTCA |  |
| *GAPDH* F | GTCTCCTCTGACTTCAACAGCG | 59°C |
| *GAPDH* R | ACCACCCTGTTGCTGTAGCCAA |  |
| *S18* F | ACCCGTTGAACCCCATTCGTGA | 59°C |
| *S18* R | GCCTCACTAAACCATCCAATCGG |  |

### *Cell proliferation, migration, and invasion assays*

Cells (2.5 × 10^5^) were split into 6-well plates and incubated in media containing 10% FBS for 6h, 12h, 24h, and 48hr. Cell proliferation was measured by Cellvia (Catalog No. LF-EZ1001; Younginfrontier, Korea). Each experiment was performed in six replicates at least three times. The cell migration analysis was studied by wound healing assay. cells (1× 10^6^) were into 6 well plates and incubated in media containing 10% FBS for 24h. Then, a straight line was made in the 6well by using a 10 μL sterile pipette tip to scratch cells. The cells were changed to containing 10% FBS and incubated 6h, 24hr, 36hr, 48hr, and 60hr. Cell invasion assays were performed in a 6.5mm Transwell with 8.0 μm Pore(Corning, USA). The transwell filter was coated with collagen type I (catalog No. A10644-01; Invitrogen). 0.5-1 ×10^5^ cells in 200μl of serum-free DMEM were seeded into the upper chamber insert. The lower chamber was filled with 0.6 ml DMEM/10% FBS. The cells at the membrane undersurface were fixed and stained after incubation for 6h, 9h, and 12h, respectively. The images were captured by light microscope (magnification, ×5).

# Supplementary Figures


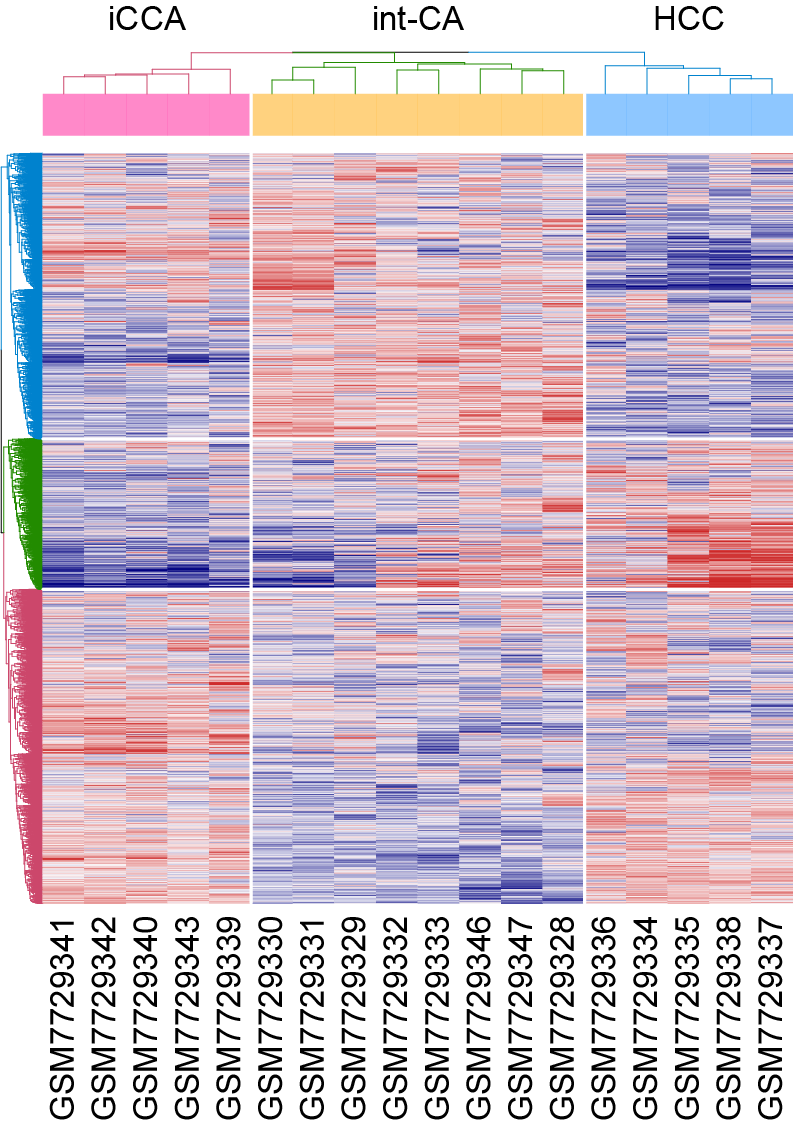


### Supplementary Figure S1. Cluster analysis of the HCC, iCCA, and int-CA transcriptome

Unsupervised hierachical clustering analysis using the variable genes (median absolute deviation > 0.5, *n* = 9,847) shows that int-CA samples are distributed in the middle between HCC and iCCA samples.

###
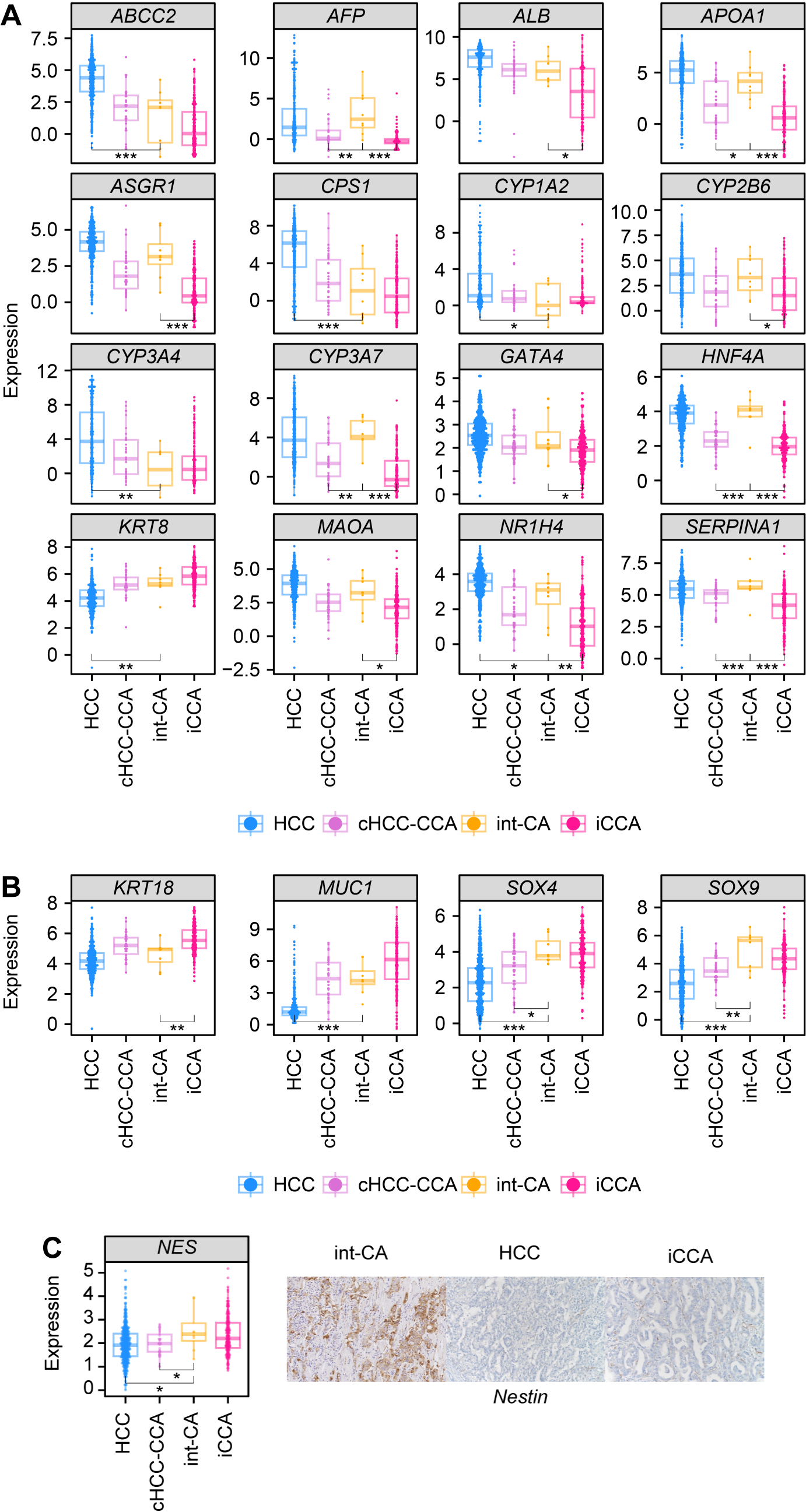


### Supplementary Figure S2. Expression of hepatic and cholangiocytic markers across tumor types

Diffrences in expression of hepatocyte and cholangiocyte marker genes across the tumor types. (A) **(**B) Boxplots show the expression level of the hepatocyte marker genes (*i.e.*, *ABCC2*, *AFP*, *ALB*, *APOA1*, *ASGR1*, *CPS1*, *CYP1A2*, *CYP2B6*, *CYP3A4*, *CYP3A7*, *GATA4*, *HNF4A*, *KRT8*, *MAOA*, *NR1H4*, and *SERPINA1*) (A) and the cholangiocyte marker genes (*i.e.*, *KRT18*, *MUC1*, *SOX4*, and *SOX9*) (B) across the tumor types. (C) Boxplot shows the expression level of the cHCC-CCA diagnostic and prognostic marker gene (*NES, right*). Immunohistochemical stain for Nestin shows a positive expression in int-CA (*right*).


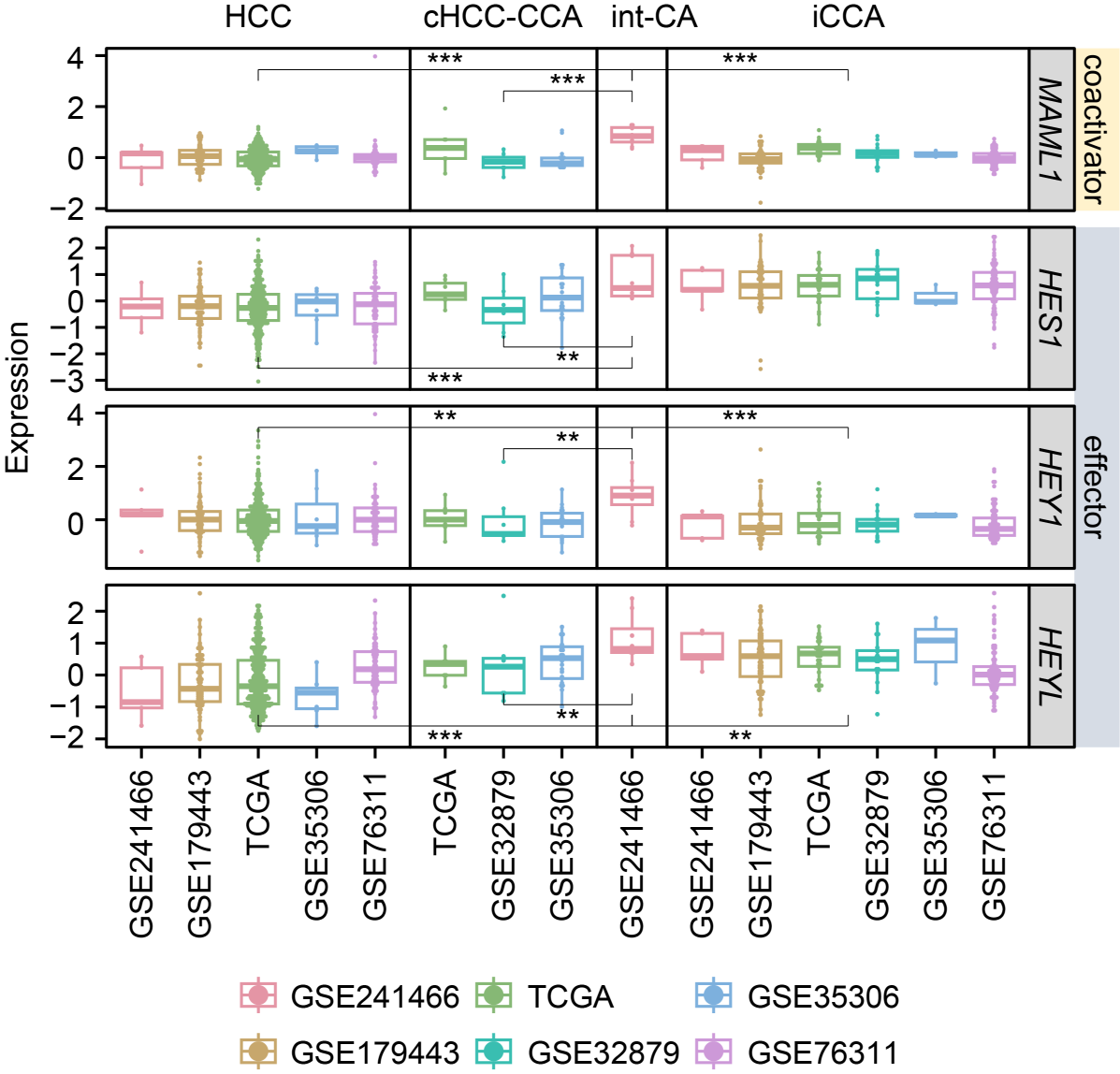


### Supplementary Figure S3. Expression of Notch coactivators and effectors

Boxplots show the expression levels of NOTCH coactivator (*MAML1*) and effectors (*i.e.*, *HES1*, *HEY1*, and *HEYL*) across the tumor types.


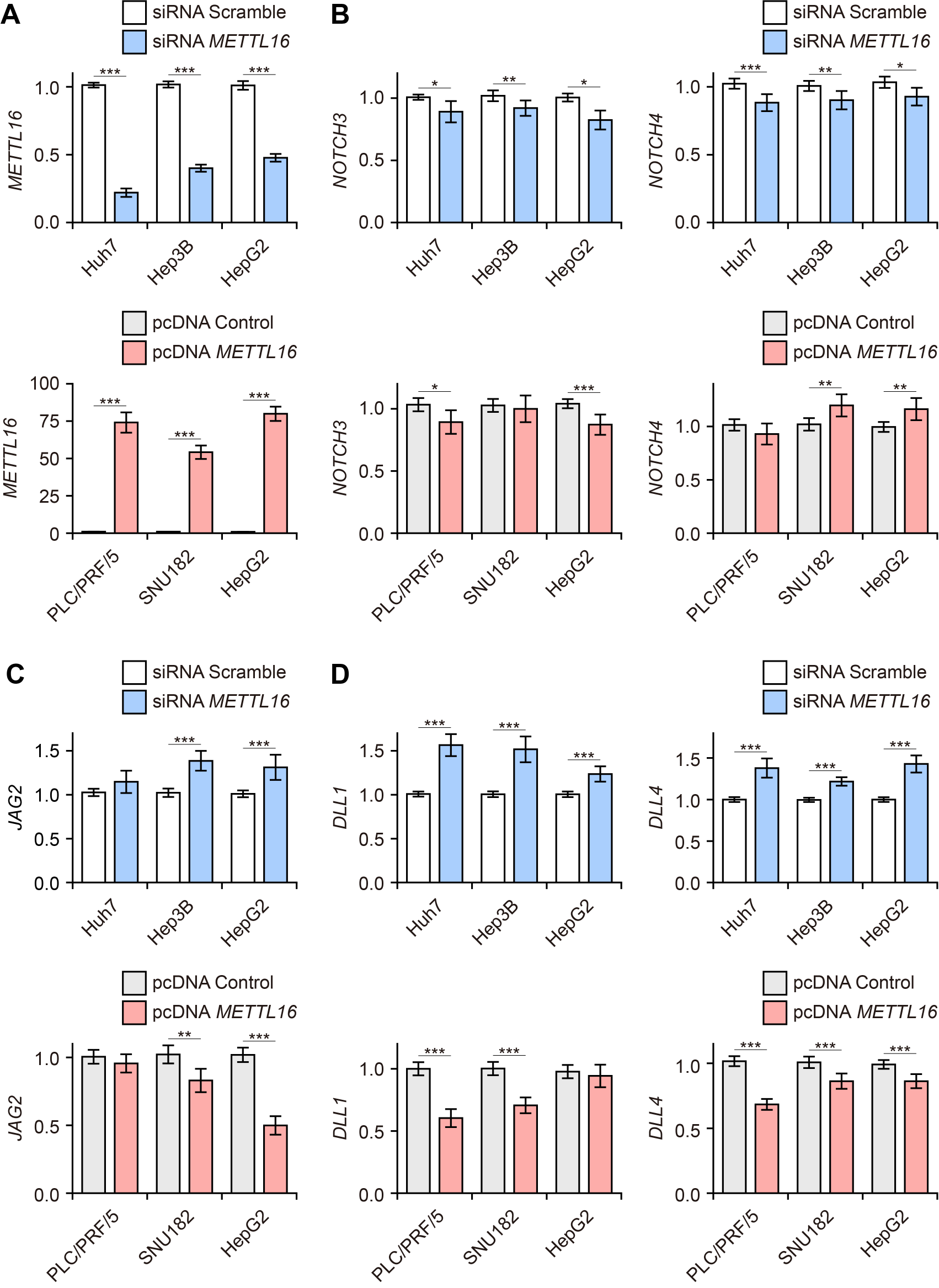


### Supplementary Figure S4. Effect of *METTL16* perturbation on Notch pathways

(A-D)**,** Effects of siRNA-mediated *METTL16* knockdown (*top*) and overexpression (*bottom*) on the expression levels of *METTL16* (A), *NOTCH3 and NOTCH4* (b), *JAG2* (**c**), DLL1 and DLL4 (**d**) are shown. The data represent the mean ± SD of 6 independent experiments. The expression values are normalized to that of GAPDH for each sample. **P* < 0.05, ***P* < 0.01, and ****P* < 0.001.


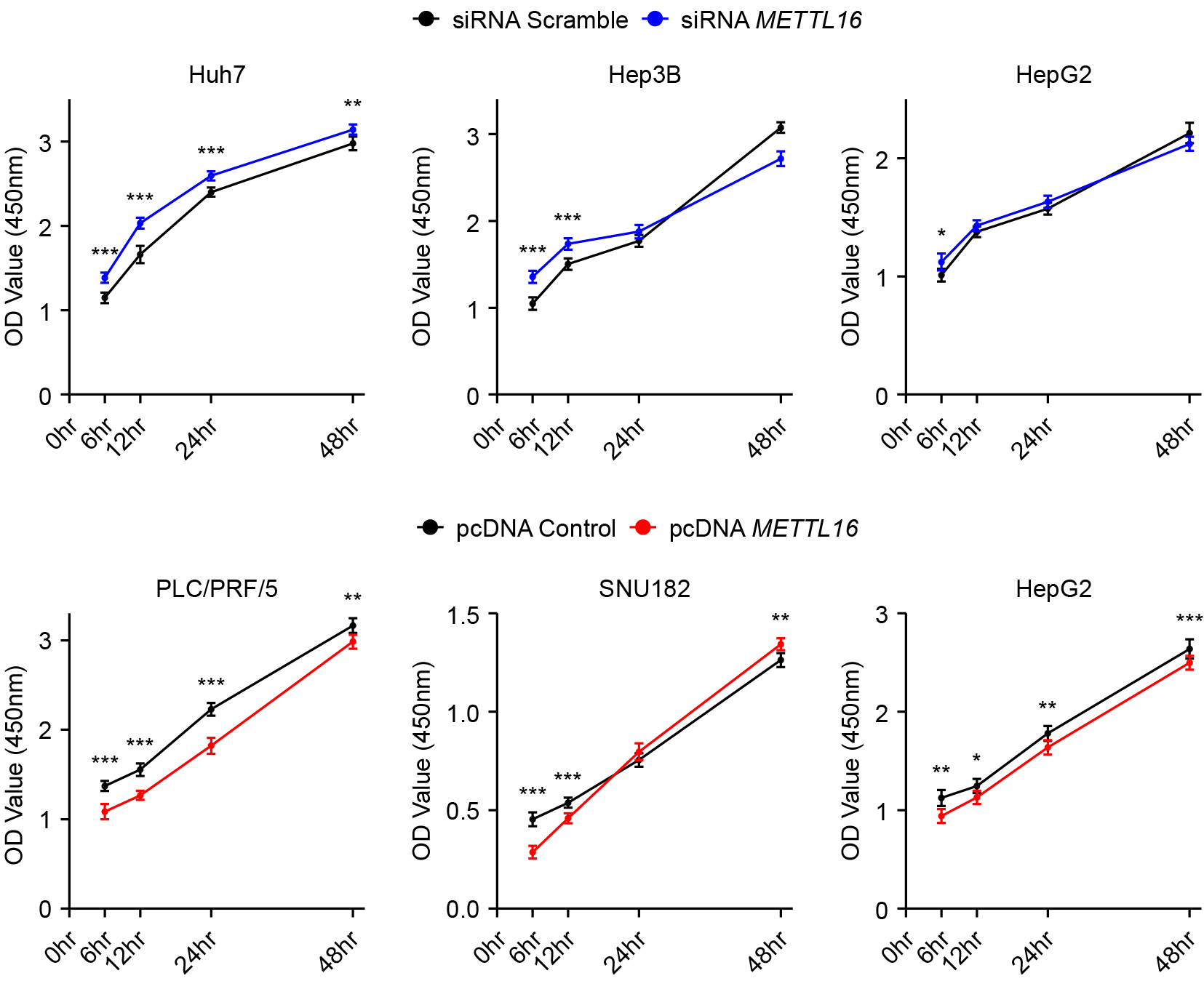


### Supplementary Figure S5. Effect of *METTL16* perturbation on cancer cell proliferation

HuH7, Hep3B, and HepG2 cells (2.5 × 10^5^ cells) are incubated in the media containing 10% FBS for the indicated times. Cell proliferation is measured by Cellvia (AbFRONTIER, LE-EZ1001A) The data represent the mean ± SD of 6 independent experiments.


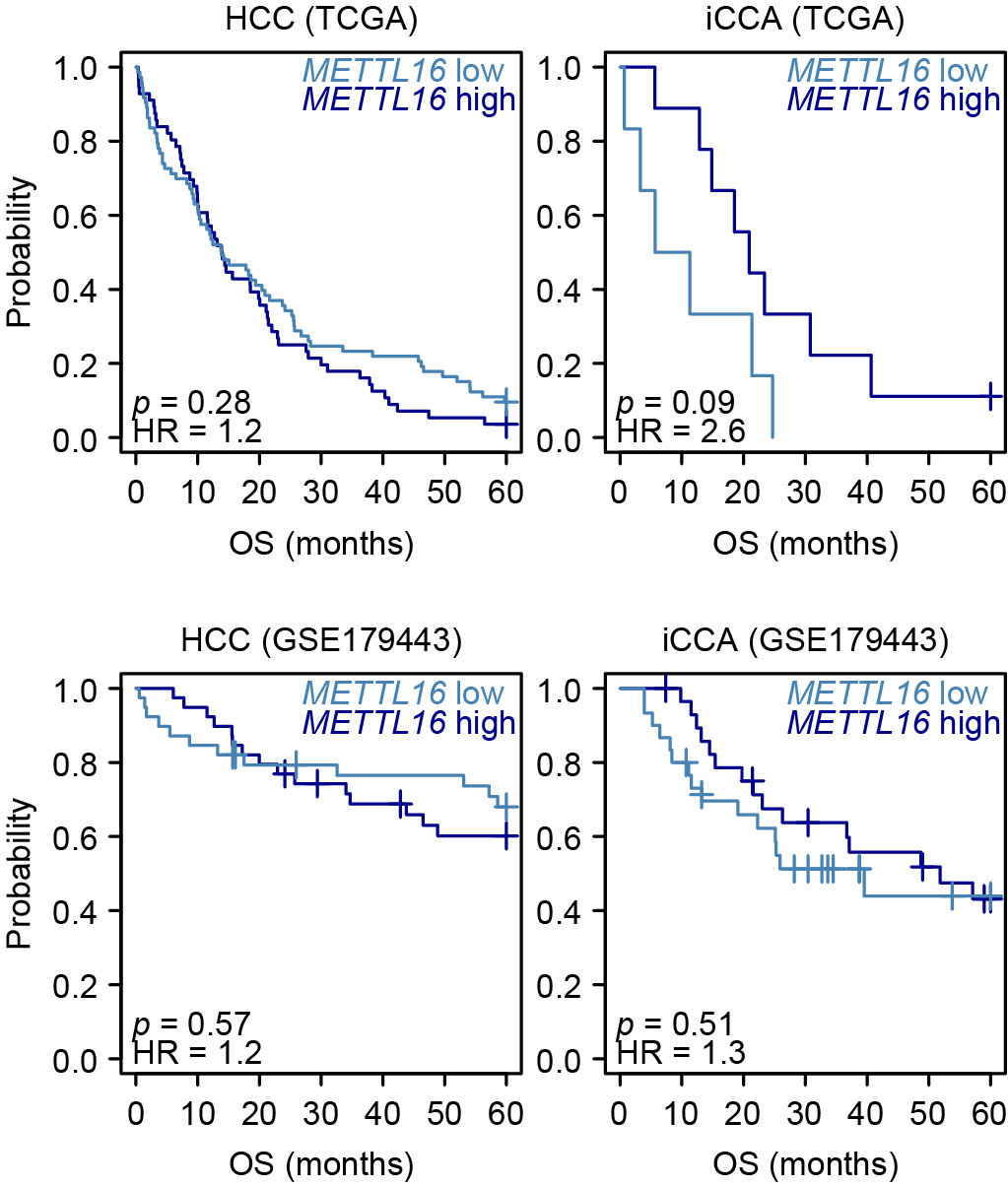


### Supplementary Figure S6. Associations of the *METTL16* expression with the prognosis of HCC or iCCA patients

Kalplan-Meire’s survival analyses for the groups of high- and low-expression of *METTL16* are shown in the data of TCGA-HCC, TCGA-iCCA, GSE179443-HCC, and GSAE179443-iCCA, respectively. The high- and low-expresssion groups for *METTL16* are stratified based on the median value of the expression levels across the samples.


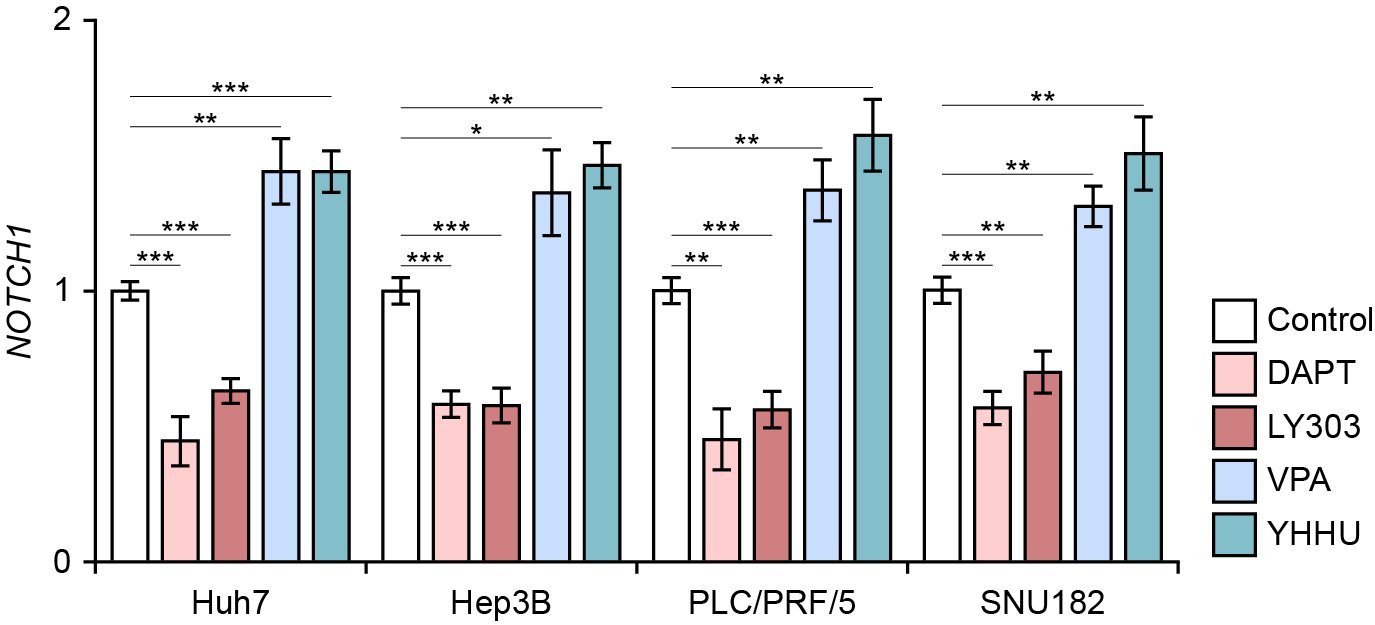


### Supplementary Figure S7. Effect of Notch inhibitors and activators on *METTL16*-mediated cancer cell invasion and migration

Notch inhibitors (DAPT, 10μM; Crenigacestat, LY303, 2nM) and Notch activators (valproic acid, VPA, 1mM; YHHU3792, YHHU, 5μM) are treated for 16hr to the indicated cells. Expression of *NOTCH1* mRNA is measured by q-PCR and normalized to GAPDH. The data represent the mean ± SD of 3 independent experiments. **P* < 0.05, ***P* < 0.01, and ****P* < 0.001.

# Supplementary Tables

### Supplementary Table S1. Clinico-pathological features of int-CA, HCC, and iCCAs

|  | int-CA (*n* = 8) | HCC (*n* = 5) | iCCA (*n* = 5) |
| --- | --- | --- | --- |
| Number of samples | 8 | 5 | 5 |
| Age (mean ± SD, yrs) | 52 ± 6 | 52 ± 6 | 58 ± 8 |
| Gender (M:F) | 7:1 | 4:1 | 4:1 |
| Risk factor (HBV/HCV/alcohol/metabolic/UK) | 8/0/0/0/0 | 3/0/0/1/1 | 1/0/0/0/4 |
| Tumor pathology |  |  |  |
| Tumor size (mean ± SD, cm) | 3.9 ± 2.2 | 3.4 ± 1.6 | 4.6 ± 1.6 |
| Microvascular invasion (%) | 5 (63 %) | 2 (40 %) | 1 (20%) |
| pT stage (no of cases) | pT1 (3), pT2 (5) | pT1 (3), pT2 (2) | pT1 (4), pT2 (1) |

Int-CA, intermediate cell carcinoma; HCC, hepatocellular carcinoma; iCCA, intrahepatic cholangiocarcinoma; SD, standard deviation; yr, years; M, male; F, female; UK, unkown

**Supplementary Table S2. Clinicopathological and follow up data of int-CA.**

| Case | Age | Sex | Tumor size  (cm) | Vascular invasion | Stage  (AJCC  5^th^ ed.) | Non-tumor liver | Etiology | Recurrrence^a^ | Death^a^ | Total follow  up duration^a^ |
| --- | --- | --- | --- | --- | --- | --- | --- | --- | --- | --- |
| 1 | 53 | M | 4.4 | Yes | pT2 | CH | HBV | Yes  (1182 days) | No | 2645 days |
| 2 | 59 | M | 5.5 | Yes | pT2 | LC | HBV | No | No | 2431 days |
| 3 | 49 | F | 2.0 | No | pT1a | LC | HBV | Yes  (976 days) | No | 2117 days |
| 4 | 50 | M | 3.7 | Yes | pT2 | CH | HBV | No | No | 1457 days |
| 5 | 46 | M | 2.5 | Yes | pT2 | LC | HBV | No | No | 4739 days |
| 6 | 68 | M | 2.8 | Yes | pT2 | CH | HBV | No | No | 2880 days |
| 7 | 62 | M | 8.7 | No | pT1b | LC | HBV | No | No | 593 days |
| 8^b^ | 46 | M | 1.5 | No | pT1a | LC | HBV | NA | Yes  (988 days) | 988 days |

M, male; F, female; CH, chronic hepatitis; LC, liver cirrhosis; HBV, hepatitis B virus.

^a^The interval between the date of operation and the date of recurrence, between the date of operation and the date of death or last follow up are expressed in days in parentheses.

^b^This patient also has multiple progressed hepatocellular carcinomas (pT2).

### Supplementary Table S3. List of the progenitor-related gene signatures

| Signature name | Description | Reference |
| --- | --- | --- |
| ESC | Embryonic stem cell | [1] |
| CLHCC | Cholangiocarcinoma-like HCC | [2] |
| HCC.stem | Stemness genes for HCC | [3-5] |
| iCCA.stem | Stemnsee genes for iCCA | [6, 7] |

### Supplementary Table S4. Differentially expressed genes (DEGs) in int-CA

| **symbol** | **Fold difference (HCC vs. others)** | ***P*-value** | **Symbol** | **Fold difference (int-CA vs. others)** | ***P*-value** | **symbol** | **Fold difference (iCCA vs. others)** | ***P*-value** |
| --- | --- | --- | --- | --- | --- | --- | --- | --- |
| **DEG for HCC** (*n* = 251) | | | **DEG for int-CA** (*n* = 132) | | | **DEG for iCCA** (*n* = 302) | | |
| *AOX1* | 4.62 | 4.7 X 10^-4^ | *FOXA3* | 2.89 | 1.5 X 10^-3^ | *CST1* | 6.54 | 2.3 X 10^-4^ |
| *GSTA2* | 4.35 | 8.2 X 10^-3^ | *ESPN* | 2.80 | 5.8 X 10^-3^ | *SPP1* | 4.68 | 6.8 X 10^-3^ |
| *ACSM2A* | 4.14 | 2.6 X 10^-3^ | *PTGDS* | 2.78 | 6.6 X 10^-3^ | *PDZK1IP1* | 4.57 | 3.3 X 10^-3^ |
| *HJV* | 4.10 | 4.7 X 10^-4^ | *PSENEN* | 2.78 | 6.4 X 10^-3^ | *TFF1* | 4.42 | 5.1 X 10^-3^ |
| *CFHR4* | 4.07 | 2.3 X 10^-3^ | *TNFRSF11B* | 2.73 | 6.6 X 10^-3^ | *TNNT1* | 4.37 | 1.4 X 10^-3^ |
| *HSD17B6* | 4.04 | 2.8 X 10^-3^ | *SALL4* | 2.63 | 9.3 X 10^-3^ | *PSORS1C1* | 4.18 | 1.2 X 10^-3^ |
| *CPS1* | 3.99 | 7.9 X 10^-3^ | *FBXO27* | 2.57 | 7.1 X 10^-3^ | *CFTR* | 4.10 | 2.3 X 10^-4^ |
| *F13B* | 3.92 | 4.7 X 10^-4^ | *TGFB3* | 2.49 | 4.5 X 10^-3^ | *ANXA3* | 4.02 | 7.0 X 10^-4^ |
| *CFHR3* | 3.79 | 1.9 X 10^-3^ | *TUBB2B* | 2.49 | 3.7 X 10^-3^ | *SYT13* | 3.96 | 2.3 X 10^-4^ |
| *CFHR2* | 3.78 | 4.2 X 10^-3^ | *PTGIS* | 2.47 | 2.7 X 10^-3^ | *RAB25* | 3.94 | 2.1 X 10^-3^ |
| *MAT1A* | 3.76 | 1.9 X 10^-3^ | *GPBAR1* | 2.41 | 5.6 X 10^-3^ | *CACNB3* | 3.88 | 1.4 X 10^-3^ |
| *KNG1* | 3.67 | 2.3 X 10^-3^ | *CCDC80* | 2.39 | 9.0 X 10^-3^ | *CLDN10* | 3.83 | 4.7 X 10^-4^ |
| *SLC47A1* | 3.64 | 1.6 X 10^-3^ | *SEC14L4* | 2.36 | 2.6 X 10^-3^ | *USH1C* | 3.82 | 4.7 X 10^-4^ |
| *UGT2B4* | 3.61 | 1.6 X 10^-3^ | *CD22* | 2.35 | 5.8 X 10^-3^ | *TCN1* | 3.80 | 7.0 X 10^-4^ |
| *APOC2* | 3.53 | 8.2 X 10^-3^ | *QSOX1* | 2.32 | 1.4 X 10^-3^ | *LAMC2* | 3.66 | 3.5 X 10^-3^ |
| *CFHR1* | 3.52 | 6.3 X 10^-3^ | *KCTD15* | 2.31 | 8.8 X 10^-3^ | *PLCD3* | 3.66 | 1.2 X 10^-3^ |
| *PLG* | 3.51 | 3.5 X 10^-3^ | *EVA1B* | 2.28 | 6.5 X 10^-3^ | *AREG* | 3.65 | 4.0 X 10^-3^ |
| *GNMT* | 3.48 | 2.1 X 10^-3^ | *ITPA* | 2.23 | 8.9 X 10^-4^ | *AC004922* | 3.64 | 2.3 X 10^-4^ |
| *CD38* | 3.48 | 8.2 X 10^-3^ | *SLC27A3* | 2.21 | 4.7 X 10^-4^ | *KRT19* | 3.62 | 2.3 X 10^-3^ |
| *ATP2B2* | 3.47 | 1.4 X 10^-3^ | *MACROH2A2* | 2.21 | 3.0 X 10^-3^ | *B3GNT3* | 3.56 | 8.6 X 10^-3^ |
| *SLCO1B1* | 3.42 | 7.0 X 10^-4^ | *ENPP2* | 2.19 | 2.6 X 10^-3^ | *FAM86B1* | 3.52 | 4.7 X 10^-3^ |
| *ITIH1* | 3.42 | 1.4 X 10^-3^ | *IGFALS* | 2.15 | 2.9 X 10^-3^ | *COMP* | 3.38 | 4.7 X 10^-3^ |
| *CDO1* | 3.39 | 4.7 X 10^-4^ | *THBS1* | 2.13 | 4.9 X 10^-3^ | *APCDD1* | 3.38 | 2.1 X 10^-3^ |
| *PLIN5* | 3.39 | 2.3 X 10^-4^ | *COL6A2* | 2.11 | 6.9 X 10^-3^ | *NPNT* | 3.30 | 7.0 X 10^-4^ |
| *AGXT* | 3.38 | 1.9 X 10^-3^ | *MMP23B* | 2.11 | 8.1 X 10^-3^ | *CLTRN* | 3.29 | 1.4 X 10^-3^ |
| *ACOX2* | 3.30 | 4.0 X 10^-3^ | *DACT1* | 2.08 | 7.5 X 10^-3^ | *CXCL8* | 3.21 | 4.7 X 10^-3^ |
| *MTARC1* | 3.3 | 2.6 X 10^-3^ | *COL15A1* | 2.07 | 1.6 X 10^-3^ | *S100A11* | 3.21 | 2.3 X 10^-4^ |
| *CCL15* | 3.28 | 9.6 X 10^-3^ | *ALDH1A3* | 2.06 | 3.2 X 10^-3^ | *LAMB3* | 3.20 | 2.6 X 10^-3^ |
| *F2* | 3.28 | 4.4 X 10^-3^ | *FBLN2* | 2.06 | 2.4 X 10^-3^ | *S100A6* | 3.15 | 1.2 X 10^-3^ |
| *CTH* | 3.23 | 5.1 X 10^-3^ | *ELP6* | 2.05 | 2.2 X 10^-3^ | *CEACAM6* | 3.12 | 9.3 X 10^-4^ |
| *CD36* | 3.18 | 7.7 X 10^-3^ | *TBXA2R* | 2.04 | 7.5 X 10^-3^ | *TFPI2* | 3.06 | 6.3 X 10^-3^ |
| *FGB* | 3.16 | 9.3 X 10^-4^ | *NOTCH3* | 2.04 | 3.0 X 10^-3^ | *MTCL1* | 3.05 | 7.2 X 10^-3^ |
| *MASP2* | 3.14 | 6.5 X 10^-3^ | *LTC4S* | 2.01 | 6.2 X 10^-3^ | *NRG3* | 3.05 | 3.5 X 10^-3^ |
| *AQP9* | 3.14 | 8.2 X 10^-3^ | *FBLIM1* | 2.00 | 4.0 X 10^-3^ | *MYEF2* | 3.03 | 4.2 X 10^-3^ |
| *ABCC2* | 3.13 | 3.0 X 10^-3^ | *TMEM160* | 1.99 | 6.1 X 10^-3^ | *GLI3* | 3.03 | 2.8 X 10^-3^ |
| *SLC38A4* | 3.10 | 4.7 X 10^-4^ | *PLTP* | 1.95 | 2.6 X 10^-3^ | *GULP1* | 3.02 | 2.3 X 10^-3^ |
| *F7* | 3.09 | 3.5 X 10^-3^ | *PODN* | 1.94 | 5.9 X 10^-3^ | *GREM1* | 3.00 | 4.4 X 10^-3^ |
| *SERPINA7* | 3.07 | 8.9 X 10^-3^ | *ALOX5* | 1.93 | 8.9 X 10^-3^ | *PKM* | 2.99 | 4.7 X 10^-4^ |
| *CYP3A7-CYP3A51P* | 3.06 | 5.4 X 10^-3^ | *FOXL1* | 1.93 | 9.7 X 10^-3^ | *ITGA3* | 2.96 | 1.9 X 10^-3^ |
| *EHHADH* | 3.02 | 9.6 X 10^-3^ | *NAV1* | 1.93 | 3.9 X 10^-3^ | *TFF2* | 2.96 | 5.4 X 10^-3^ |
| *ITIH3* | 3.01 | 3.5 X 10^-3^ | *PRSS23* | 1.92 | 6.0 X 10^-3^ | *EDA* | 2.90 | 9.3 X 10^-4^ |
| *ETNPPL* | 3.01 | 3.3 X 10^-3^ | *EMILIN1* | 1.91 | 2.4 X 10^-3^ | *TSPAN1* | 2.89 | 2.6 X 10^-3^ |
| *TPRG1* | 2.96 | 2.3 X 10^-3^ | *COL6A3* | 1.88 | 8.7 X 10^-3^ | *NTM* | 2.88 | 1.4 X 10^-3^ |
| *SLC6A1* | 2.92 | 2.1 X 10^-3^ | *GSTT2B* | 1.87 | 7.5 X 10^-3^ | *SPEG* | 2.87 | 4.7 X 10^-3^ |
| *ADH1A* | 2.91 | 5.6 X 10^-3^ | *LRRC32* | 1.87 | 6.7 X 10^-3^ | *TUBB3* | 2.83 | 2.3 X 10^-4^ |
| *UGT2B10* | 2.89 | 7.7 X 10^-3^ | *DOCK6* | 1.84 | 2.6 X 10^-3^ | *NLRP2* | 2.82 | 7.0 X 10^-4^ |
| *PLPPR1* | 2.88 | 2.8 X 10^-3^ | *DDX51* | 1.84 | 5.1 X 10^-3^ | *TM4SF1* | 2.77 | 4.9 X 10^-3^ |
| *RGN* | 2.88 | 4.7 X 10^-3^ | *TBX2* | 1.84 | 1.9 X 10^-3^ | *CLDN11* | 2.73 | 2.6 X 10^-3^ |
| *C8A* | 2.87 | 8.9 X 10^-3^ | *FHL1* | 1.83 | 2.9 X 10^-3^ | *DDR1* | 2.72 | 2.3 X 10^-4^ |
| *AS3MT* | 2.80 | 2.6 X 10^-3^ | *SHISA3* | 1.83 | 3.1 X 10^-3^ | *SEMA3E* | 2.69 | 6.1 X 10^-3^ |
| *ORM2* | 2.75 | 6.1 X 10^-3^ | *TCF4* | 1.81 | 3.9 X 10^-3^ | *DNM1* | 2.69 | 2.3 X 10^-4^ |
| *FGG* | 2.73 | 3.7 X 10^-3^ | *PXDN* | 1.80 | 8.2 X 10^-3^ | *PFKP* | 2.68 | 4.7 X 10^-4^ |
| *PAH* | 2.72 | 9.3 X 10^-4^ | *PEPD* | 1.79 | 4.3 X 10^-3^ | *CSGALNACT1* | 2.68 | 1.6 X 10^-3^ |
| *SCUBE1* | 2.66 | 5.4 X 10^-3^ | *MCAM* | 1.76 | 6.6 X 10^-3^ | *CARD11* | 2.66 | 2.6 X 10^-3^ |
| *GGH* | 2.64 | 6.1 X 10^-3^ | *NEK8* | 1.74 | 9.7 X 10^-3^ | *SULT2B1* | 2.66 | 1.4 X 10^-3^ |
| *CECR2* | 2.63 | 2.8 X 10^-3^ | *CYBRD1* | 1.74 | 4.8 X 10^-3^ | *F3* | 2.63 | 4.0 X 10^-3^ |
| *FGA* | 2.62 | 5.1 X 10^-3^ | *EXOC6* | 1.72 | 1.8 X 10^-3^ | *LPCAT4* | 2.62 | 1.6 X 10^-3^ |
| *TCAIM* | 2.59 | 2.3 X 10^-4^ | *ADAMTS10* | 1.71 | 6.3 X 10^-3^ | *MMP11* | 2.62 | 5.8 X 10^-3^ |
| *LCMT1* | 2.58 | 2.6 X 10^-3^ | *ERG* | 1.71 | 4.4 X 10^-3^ | *ITGB8* | 2.59 | 2.1 X 10^-3^ |
| *LIPC* | 2.57 | 5.1 X 10^-3^ | *CDKN1C* | 1.69 | 7.3 X 10^-3^ | *CASTOR3P* | 2.58 | 4.2 X 10^-3^ |
| *LCN12* | 2.56 | 2.3 X 10^-3^ | *LHX2* | 1.67 | 4.2 X 10^-3^ | *ITGA2* | 2.58 | 2.3 X 10^-4^ |
| *TSHR* | 2.56 | 7.0 X 10^-4^ | *NENF* | 1.65 | 6.2 X 10^-3^ | *TNFRSF10C* | 2.55 | 4.7 X 10^-3^ |
| *TM7SF2* | 2.55 | 4.7 X 10^-4^ | *TNFRSF6B* | 1.64 | 4.4 X 10^-3^ | *OLR1* | 2.53 | 8.2 X 10^-3^ |
| *SLC17A2* | 2.55 | 3.3 X 10^-3^ | *ECE1* | 1.64 | 3.6 X 10^-3^ | *CAPRIN2* | 2.52 | 2.3 X 10^-4^ |
| *BNIP3* | 2.42 | 2.3 X 10^-4^ | *ARID5B* | 1.64 | 6.3 X 10^-3^ | *BNIP3L* | 2.51 | 2.3 X 10^-4^ |
| *MAOA* | 2.41 | 5.8 X 10^-3^ | *BPIFB2* | 1.63 | 8.8 X 10^-3^ | *ADAM28* | 2.50 | 4.0 X 10^-3^ |
| *UBE2D4* | 2.39 | 2.6 X 10^-3^ | *NRM* | 1.60 | 9.7 X 10^-3^ | *GSDME* | 2.45 | 5.4 X 10^-3^ |
| *ZNF563* | 2.39 | 4.7 X 10^-4^ | *RTN1* | 1.58 | 7.9 X 10^-3^ | *SMPD3* | 2.45 | 7.0 X 10^-4^ |
| *F11* | 2.39 | 5.8 X 10^-3^ | *STING1* | 1.57 | 2.1 X 10^-3^ | *GALNT3* | 2.44 | 6.1 X 10^-3^ |
| *RNF123* | 2.37 | 8.2 X 10^-3^ | *NBEAL2* | 1.57 | 9.3 X 10^-3^ | *HAND2* | 2.44 | 7.0 X 10^-4^ |
| *HGD* | 2.35 | 9.1 X 10^-3^ | *CDK18* | 1.54 | 5.7 X 10^-3^ | *GALNT7* | 2.43 | 3.7 X 10^-3^ |
| *ACADSB* | 2.32 | 8.6 X 10^-3^ | *PTK6* | 1.53 | 2.4 X 10^-3^ | *TNFRSF21* | 2.42 | 7.0 X 10^-4^ |
| *DAPK2* | 2.32 | 2.1 X 10^-3^ | *JCAD* | 1.52 | 1.8 X 10^-3^ | *TANC2* | 2.42 | 5.4 X 10^-3^ |
| *MST1* | 2.31 | 9.6 X 10^-3^ | *BIN1* | 1.52 | 2.2 X 10^-3^ | *CST4* | 2.41 | 4.7 X 10^-4^ |
| *MTRF1L* | 2.30 | 9.3 X 10^-4^ | *ATXN10* | 1.50 | 6.1 X 10^-4^ | *SLC25A36* | 2.37 | 2.3 X 10^-4^ |
| *TCEA3* | 2.26 | 3.7 X 10^-3^ | *LRRK1* | 1.49 | 7.9 X 10^-3^ | *ODF2L* | 2.35 | 1.4 X 10^-3^ |
| *CYP3A43* | 2.26 | 2.3 X 10^-4^ | *SUSD2* | 1.48 | 4.9 X 10^-3^ | *PDGFD* | 2.34 | 5.6 X 10^-3^ |
| *ACSS3* | 2.25 | 1.4 X 10^-3^ | *BAP1* | 1.48 | 9.3 X 10^-3^ | *AKT3* | 2.32 | 2.1 X 10^-3^ |
| *ECI2* | 2.24 | 2.3 X 10^-4^ | *COL18A1* | 1.48 | 3.4 X 10^-3^ | *BCL9L* | 2.32 | 1.2 X 10^-3^ |
| *IDI1* | 2.24 | 1.6 X 10^-3^ | *RGS5* | 1.48 | 8.4 X 10^-3^ | *TNFRSF11A* | 2.29 | 9.3 X 10^-4^ |
| *C5* | 2.21 | 4.2 X 10^-3^ | *HSPG2* | 1.46 | 2.8 X 10^-3^ | *PAM* | 2.28 | 1.6 X 10^-3^ |
| *SEC16B* | 2.19 | 1.9 X 10^-3^ | *COL6A1* | 1.46 | 4.6 X 10^-3^ | *ATP6V1F* | 2.26 | 4.7 X 10^-4^ |
| *DHRS1* | 2.19 | 5.6 X 10^-3^ | *IFNGR2* | 1.45 | 8.0 X 10^-3^ | *S100A4* | 2.26 | 5.4 X 10^-3^ |
| *BMAL1* | 2.19 | 4.9 X 10^-3^ | *TMSB4X* | 1.45 | 4.2 X 10^-3^ | *NPR3* | 2.26 | 5.1 X 10^-3^ |
| *FAM184A* | 2.17 | 8.6 X 10^-3^ | *CIC* | 1.45 | 2.1 X 10^-3^ | *TC2N* | 2.26 | 3.0 X 10^-3^ |
| *NADK2* | 2.16 | 5.6 X 10^-3^ | *COG8* | 1.45 | 1.7 X 10^-3^ | *MSLN* | 2.24 | 4.7 X 10^-4^ |
| *ALDH5A1* | 2.15 | 6.8 X 10^-3^ | *PRSS50* | 1.45 | 8.5 X 10^-3^ | *PRSS16* | 2.23 | 1.2 X 10^-3^ |
| *NOS1AP* | 2.15 | 3.7 X 10^-3^ | *NOTCH4* | 1.44 | 7.7 X 10^-3^ | *GPATCH11* | 2.23 | 9.3 X 10^-4^ |
| *SLC22A9* | 2.14 | 6.8 X 10^-3^ | *KDM6B* | 1.43 | 5.7 X 10^-3^ | *GPSM2* | 2.22 | 2.3 X 10^-3^ |
| *SLC10A5* | 2.14 | 2.3 X 10^-4^ | *ENTPD6* | 1.43 | 1.6 X 10^-3^ | *DBN1* | 2.21 | 4.7 X 10^-4^ |
| *MOCS2* | 2.14 | 6.1 X 10^-3^ | *UPF1* | 1.43 | 5.6 X 10^-3^ | *AFF2* | 2.21 | 2.1 X 10^-3^ |
| *F5* | 2.13 | 3.5 X 10^-3^ | *CPXM2* | 1.42 | 6.4 X 10^-3^ | *SCRN1* | 2.21 | 5.1 X 10^-3^ |
| *UGDH* | 2.12 | 4.2 X 10^-3^ | *POFUT1* | 1.40 | 8.6 X 10^-3^ | *DNASE1* | 2.21 | 8.2 X 10^-3^ |
| *DTX3L* | 2.11 | 4.7 X 10^-4^ | *SIPA1L2* | 1.39 | 6.1 X 10^-3^ | *SRI* | 2.20 | 4.7 X 10^-4^ |
| *KLKB1* | 2.10 | 6.5 X 10^-3^ | *RRP7A* | 1.37 | 5.7 X 10^-3^ | *GALNT10* | 2.19 | 3.7 X 10^-3^ |
| *KLF15* | 2.08 | 4.7 X 10^-3^ | *SELENON* | 1.35 | 5.2 X 10^-3^ | *S100A10* | 2.19 | 7.9 X 10^-3^ |
| *PLIN4* | 2.08 | 1.6 X 10^-3^ | *MPZL2* | 1.35 | 9.9 X 10^-3^ | *PTPN13* | 2.19 | 9.3 X 10^-3^ |
| *PSAT1* | 2.08 | 2.6 X 10^-3^ | *BCOR* | 1.34 | 3.4 X 10^-3^ | *SLCO4A1* | 2.18 | 2.3 X 10^-3^ |
| *CLYBL* | 2.07 | 1.2 X 10^-3^ | *MAB21L4* | 1.34 | 6.0 X 10^-3^ | *KRCC1* | 2.17 | 2.3 X 10^-4^ |
| *PI4K2B* | 2.07 | 9.3 X 10^-4^ | *NAGA* | 1.34 | 1.6 X 10^-3^ | *KIF3A* | 2.17 | 2.3 X 10^-3^ |
| *TMEM150B* | 2.06 | 8.2 X 10^-3^ | *BTG2* | 1.32 | 8.2 X 10^-3^ | *GABBR1* | 2.15 | 5.8 X 10^-3^ |
| *CUX2* | 2.06 | 3.3 X 10^-3^ | *TCF7L1* | 1.32 | 6.3 X 10^-3^ | *TNC* | 2.15 | 9.3 X 10^-4^ |
| *RABEPK* | 2.05 | 1.2 X 10^-3^ | *JAK3* | 1.31 | 8.8 X 10^-3^ | *SLC16A3* | 2.15 | 4.0 X 10^-3^ |
| *TLCD4* | 2.05 | 3.5 X 10^-3^ | *NOTCH1* | 1.31 | 3.3 X 10^-3^ | *LAYN* | 2.15 | 1.4 X 10^-3^ |
| *TTC38* | 2.02 | 7.5 X 10^-3^ | *STAB1* | 1.30 | 9.0 X 10^-3^ | *CLIP2* | 2.15 | 2.3 X 10^-3^ |
| *GSTZ1* | 2.01 | 6.3 X 10^-3^ | *MARK4* | 1.29 | 8.6 X 10^-3^ | *GSTP1* | 2.14 | 3.7 X 10^-3^ |
| *MSH5-SAPCD1* | 2.00 | 2.8 X 10^-3^ | *RTKN* | 1.28 | 5.2 X 10^-3^ | *GBP3* | 2.13 | 6.5 X 10^-3^ |
| *HSDL2* | 2.00 | 1.2 X 10^-3^ | *TIMP3* | 1.28 | 4.4 X 10^-3^ | *TES* | 2.13 | 1.6 X 10^-3^ |
| *CLTCL1* | 1.99 | 8.2 X 10^-3^ | *TBC1D10A* | 1.28 | 8.8 X 10^-3^ | *SLC38A1* | 2.12 | 2.1 X 10^-3^ |
| *ITPR2* | 1.98 | 4.0 X 10^-3^ | *FRMD4A* | 1.27 | 3.0 X 10^-3^ | *SLC6A6* | 2.11 | 9.3 X 10^-3^ |
| *TMED5* | 1.97 | 2.6 X 10^-3^ | *EP300* | 1.24 | 2.3 X 10^-3^ | *ITPR3* | 2.11 | 2.8 X 10^-3^ |
| *PPIF* | 1.97 | 1.6 X 10^-3^ | *PPP2R1A* | 1.23 | 4.9 X 10^-3^ | *MNT* | 2.10 | 4.7 X 10^-4^ |
| *ABHD6* | 1.96 | 6.8 X 10^-3^ | *SORBS3* | 1.22 | 7.4 X 10^-3^ | *FRMD6* | 2.10 | 2.8 X 10^-3^ |
| *AURKB* | 1.95 | 3.0 X 10^-3^ | *CNNM3* | 1.21 | 9.7 X 10^-3^ | *ARMC9* | 2.09 | 2.3 X 10^-3^ |
| *PLA2G7* | 1.95 | 2.3 X 10^-4^ | *PLBD2* | 1.21 | 6.1 X 10^-3^ | *PRTFDC1* | 2.08 | 1.4 X 10^-3^ |
| *NDUFS3* | 1.95 | 8.6 X 10^-3^ | *ARHGEF1* | 1.20 | 9.3 X 10^-3^ | *PCNX2* | 2.08 | 1.6 X 10^-3^ |
| *WNK3* | 1.93 | 9.3 X 10^-3^ | *TMEM109* | 1.18 | 3.3 X 10^-3^ | *RALA* | 2.08 | 7.0 X 10^-4^ |
| *TMEM161A* | 1.92 | 2.6 X 10^-3^ | *JMJD8* | 1.17 | 2.3 X 10^-3^ | *THSD4* | 2.07 | 9.3 X 10^-3^ |
| *ATP5MJ* | 1.91 | 7.0 X 10^-4^ | *DHRS7* | 1.17 | 8.9 X 10^-3^ | *PCSK1N* | 2.07 | 9.3 X 10^-3^ |
| *ERO1B* | 1.90 | 9.3 X 10^-3^ | *GNAS* | 1.17 | 5.6 X 10^-3^ | *ENKD1* | 2.06 | 2.1 X 10^-3^ |
| *NAGS* | 1.90 | 7.7 X 10^-3^ | *ZNF281* | 1.16 | 2.2 X 10^-3^ | *TUSC3* | 2.06 | 7.7 X 10^-3^ |
| *UBXN11* | 1.87 | 6.3 X 10^-3^ | *OXT* | 1.15 | 3.3 X 10^-4^ | *AMH* | 2.06 | 2.8 X 10^-3^ |
| *FAM149B1* | 1.85 | 2.1 X 10^-3^ | *MAN2B1* | 1.15 | 4.0 X 10^-3^ | *PRRX1* | 2.06 | 6.5 X 10^-3^ |
| *BUD31* | 1.84 | 7.0 X 10^-4^ | *SELENOW* | 1.15 | 7.0 X 10^-3^ | *HK2* | 2.05 | 1.9 X 10^-3^ |
| *TRMT11* | 1.83 | 4.7 X 10^-3^ | *HAS1* | 1.09 | 3.9 X 10^-3^ | *SNX10* | 2.03 | 8.9 X 10^-3^ |
| *STAG1* | 1.83 | 9.3 X 10^-4^ | *SOCS1* | 1.07 | 6.0 X 10^-3^ | *PEF1* | 2.02 | 7.2 X 10^-3^ |
| *THBS4* | 1.81 | 7.9 X 10^-3^ | *FLCN* | 1.06 | 5.8 X 10^-3^ | *CHST7* | 2.01 | 9.8 X 10^-3^ |
| *POLB* | 1.80 | 1.2 X 10^-3^ | *KCNK17* | 1.05 | 8.1 X 10^-3^ | *RPGRIP1L* | 2.00 | 1.9 X 10^-3^ |
| *OASL* | 1.79 | 8.2 X 10^-3^ | *RALY* | 1.03 | 5.8 X 10^-3^ | *SEMA3B* | 1.99 | 7.5 X 10^-3^ |
| *ADPRHL1* | 1.79 | 9.3 X 10^-4^ | *CLIC4* | 1.02 | 6.9 X 10^-3^ | *RELCH* | 1.99 | 3.0 X 10^-3^ |
| *UQCR11* | 1.79 | 4.2 X 10^-3^ | *ATP9A* | 1.02 | 3.0 X 10^-3^ | *UGT8* | 1.98 | 1.2 X 10^-3^ |
| *MPC1* | 1.78 | 1.2 X 10^-3^ | *TNNT3* | 1.00 | 3.2 X 10^-3^ | *ABR* | 1.98 | 2.6 X 10^-3^ |
| *CAPN3* | 1.75 | 3.0 X 10^-3^ |  |  |  | *DOC2A* | 1.97 | 9.3 X 10^-4^ |
| *DEPDC7* | 1.74 | 5.8 X 10^-3^ |  |  |  | *DCBLD2* | 1.97 | 2.3 X 10^-4^ |
| *GLRX2* | 1.74 | 2.3 X 10^-4^ |  |  |  | *ZNF512* | 1.97 | 9.3 X 10^-4^ |
| *COMMD10* | 1.72 | 2.1 X 10^-3^ |  |  |  | *FAR1* | 1.96 | 2.3 X 10^-4^ |
| *MRPS26* | 1.72 | 1.9 X 10^-3^ |  |  |  | *CALU* | 1.96 | 6.8 X 10^-3^ |
| *ATPSCKMT* | 1.71 | 6.1 X 10^-3^ |  |  |  | *SGPP2* | 1.96 | 1.4 X 10^-3^ |
| *ARG2* | 1.71 | 7.7 X 10^-3^ |  |  |  | *ID1* | 1.96 | 7.7 X 10^-3^ |
| *AIFM2* | 1.71 | 4.9 X 10^-3^ |  |  |  | *DMTN* | 1.95 | 7.0 X 10^-4^ |
| *MMUT* | 1.69 | 2.3 X 10^-3^ |  |  |  | *CLUAP1* | 1.94 | 1.4 X 10^-3^ |
| *AMACR* | 1.68 | 8.6 X 10^-3^ |  |  |  | *CCDC186* | 1.93 | 7.2 X 10^-3^ |
| *ACSS2* | 1.68 | 9.6 X 10^-3^ |  |  |  | *ARFIP1* | 1.92 | 3.5 X 10^-3^ |
| *RBPMS2* | 1.67 | 1.6 X 10^-3^ |  |  |  | *AFAP1L2* | 1.91 | 9.3 X 10^-4^ |
| *MRPL4* | 1.66 | 5.4 X 10^-3^ |  |  |  | *TEX9* | 1.90 | 3.0 X 10^-3^ |
| *EMC6* | 1.66 | 6.3 X 10^-3^ |  |  |  | *SCAMP1* | 1.90 | 7.0 X 10^-4^ |
| *NR1H3* | 1.65 | 7.0 X 10^-3^ |  |  |  | *BORCS5* | 1.89 | 8.6 X 10^-3^ |
| *MLANA* | 1.65 | 9.1 X 10^-3^ |  |  |  | *SMAD3* | 1.88 | 3.5 X 10^-3^ |
| *GLRX5* | 1.65 | 7.7 X 10^-3^ |  |  |  | *FIBCD1* | 1.85 | 4.2 X 10^-3^ |
| *CAMK1* | 1.64 | 7.2 X 10^-3^ |  |  |  | *CCSER1* | 1.84 | 3.0 X 10^-3^ |
| *SLC25A33* | 1.62 | 2.3 X 10^-4^ |  |  |  | *ZNF827* | 1.83 | 5.6 X 10^-3^ |
| *TFAP4* | 1.62 | 4.9 X 10^-3^ |  |  |  | *ACP3* | 1.82 | 2.1 X 10^-3^ |
| *NUPR1* | 1.61 | 1.9 X 10^-3^ |  |  |  | *SLC37A3* | 1.82 | 2.3 X 10^-4^ |
| *RABGGTA* | 1.61 | 8.9 X 10^-3^ |  |  |  | *CCDC74B* | 1.81 | 4.7 X 10^-3^ |
| *NFYB* | 1.60 | 8.9 X 10^-3^ |  |  |  | *KRT15* | 1.80 | 4.7 X 10^-3^ |
| *MTFR1* | 1.60 | 4.9 X 10^-3^ |  |  |  | *IDS* | 1.79 | 2.3 X 10^-4^ |
| *GFRA1* | 1.60 | 5.4 X 10^-3^ |  |  |  | *USP8* | 1.78 | 3.5 X 10^-3^ |
| *ABCC9* | 1.60 | 2.3 X 10^-3^ |  |  |  | *BCL2L1* | 1.77 | 1.2 X 10^-3^ |
| *ELOA* | 1.60 | 1.2 X 10^-3^ |  |  |  | *SPECC1* | 1.76 | 9.6 X 10^-3^ |
| *EPB41L4B* | 1.58 | 2.3 X 10^-3^ |  |  |  | *MYOF* | 1.76 | 4.7 X 10^-4^ |
| *SUGCT* | 1.57 | 5.4 X 10^-3^ |  |  |  | *IQGAP1* | 1.73 | 1.2 X 10^-3^ |
| *TEX30* | 1.57 | 9.3 X 10^-4^ |  |  |  | *HOPX* | 1.73 | 5.6 X 10^-3^ |
| *RTP3* | 1.57 | 8.4 X 10^-3^ |  |  |  | *GCC2* | 1.73 | 4.4 X 10^-3^ |
| *NDUFB9* | 1.56 | 6.1 X 10^-3^ |  |  |  | *LPIN3* | 1.72 | 7.5 X 10^-3^ |
| *SRXN1* | 1.56 | 2.3 X 10^-4^ |  |  |  | *FRMD5* | 1.72 | 7.2 X 10^-3^ |
| *NEDD4* | 1.55 | 4.7 X 10^-4^ |  |  |  | *CTAGE15* | 1.71 | 2.3 X 10^-3^ |
| *LONP1* | 1.55 | 9.3 X 10^-4^ |  |  |  | *EPHA6* | 1.71 | 6.3 X 10^-3^ |
| *NCOA2* | 1.54 | 5.8 X 10^-3^ |  |  |  | *LUC7L* | 1.70 | 2.6 X 10^-3^ |
| *METAP2* | 1.54 | 6.5 X 10^-3^ |  |  |  | *SEL1L3* | 1.70 | 4.2 X 10^-3^ |
| *ASPH* | 1.53 | 1.6 X 10^-3^ |  |  |  | *PLCH1* | 1.69 | 7.0 X 10^-4^ |
| *NOP58* | 1.53 | 2.1 X 10^-3^ |  |  |  | *TPM1* | 1.69 | 7.0 X 10^-3^ |
| *OCEL1* | 1.52 | 5.6 X 10^-3^ |  |  |  | *MORF4L1* | 1.69 | 1.4 X 10^-3^ |
| *TATDN3* | 1.52 | 9.6 X 10^-3^ |  |  |  | *SPIN2A* | 1.67 | 4.7 X 10^-4^ |
| *EBAG9* | 1.52 | 2.6 X 10^-3^ |  |  |  | *AP1S3* | 1.67 | 1.9 X 10^-3^ |
| *TRIM27* | 1.51 | 4.7 X 10^-4^ |  |  |  | *TMEM87B* | 1.67 | 3.5 X 10^-3^ |
| *POLN* | 1.49 | 4.0 X 10^-3^ |  |  |  | *MEIS3* | 1.67 | 8.6 X 10^-3^ |
| *SGTB* | 1.47 | 1.9 X 10^-3^ |  |  |  | *DPP10* | 1.65 | 6.3 X 10^-3^ |
| *MRPL40* | 1.47 | 4.9 X 10^-3^ |  |  |  | *SERF1B* | 1.65 | 5.4 X 10^-3^ |
| *CEP43* | 1.45 | 2.3 X 10^-4^ |  |  |  | *PFKM* | 1.64 | 3.3 X 10^-3^ |
| *TMEM245* | 1.45 | 1.2 X 10^-3^ |  |  |  | *TRIP10* | 1.63 | 6.8 X 10^-3^ |
| *PEX16* | 1.44 | 5.6 X 10^-3^ |  |  |  | *DGKI* | 1.63 | 2.8 X 10^-3^ |
| *ZHX1* | 1.43 | 2.6 X 10^-3^ |  |  |  | *RPS6KA2* | 1.61 | 5.8 X 10^-3^ |
| *TMEM14A* | 1.43 | 2.1 X 10^-3^ |  |  |  | *ZRSR2* | 1.61 | 9.8 X 10^-3^ |
| *CPOX* | 1.42 | 5.6 X 10^-3^ |  |  |  | *SMIM15* | 1.61 | 7.2 X 10^-3^ |
| *ANAPC10* | 1.42 | 9.1 X 10^-3^ |  |  |  | *GMIP* | 1.60 | 6.1 X 10^-3^ |
| *TAF13* | 1.42 | 3.7 X 10^-3^ |  |  |  | *MAGOHB* | 1.60 | 4.7 X 10^-3^ |
| *KEAP1* | 1.42 | 7.9 X 10^-3^ |  |  |  | *ARMCX2* | 1.60 | 4.7 X 10^-4^ |
| *ZNF680* | 1.41 | 2.6 X 10^-3^ |  |  |  | *ZNF117* | 1.60 | 1.2 X 10^-3^ |
| *LRRC8D* | 1.41 | 7.5 X 10^-3^ |  |  |  | *SAMD9* | 1.60 | 1.9 X 10^-3^ |
| *CTNNAL1* | 1.41 | 5.8 X 10^-3^ |  |  |  | *STK32A* | 1.59 | 9.3 X 10^-4^ |
| *RMND1* | 1.39 | 3.7 X 10^-3^ |  |  |  | *CTAGE6* | 1.59 | 9.3 X 10^-4^ |
| *BLK* | 1.39 | 5.4 X 10^-3^ |  |  |  | *AKR1B1* | 1.59 | 2.6 X 10^-3^ |
| *ZNF44* | 1.39 | 9.3 X 10^-4^ |  |  |  | *CORO2B* | 1.59 | 3.5 X 10^-3^ |
| *TXNRD3* | 1.38 | 7.0 X 10^-4^ |  |  |  | *CYB5B* | 1.58 | 8.9 X 10^-3^ |
| *C2CD4D* | 1.36 | 3.7 X 10^-3^ |  |  |  | *RAP2C* | 1.57 | 2.3 X 10^-3^ |
| *SLC46A3* | 1.36 | 8.4 X 10^-3^ |  |  |  | *ADAP2* | 1.56 | 2.1 X 10^-3^ |
| *STPG3* | 1.36 | 6.1 X 10^-3^ |  |  |  | *CFAP97* | 1.56 | 7.7 X 10^-3^ |
| *ERLIN1* | 1.35 | 6.8 X 10^-3^ |  |  |  | *ARL14* | 1.55 | 1.6 X 10^-3^ |
| *ZNF280C* | 1.34 | 8.6 X 10^-3^ |  |  |  | *KLK11* | 1.54 | 4.7 X 10^-4^ |
| *SDHC* | 1.33 | 7.2 X 10^-3^ |  |  |  | *OSBPL1A* | 1.54 | 8.6 X 10^-3^ |
| *CIPC* | 1.33 | 8.6 X 10^-3^ |  |  |  | *FMNL2* | 1.54 | 6.1 X 10^-3^ |
| *MTMR4* | 1.32 | 8.2 X 10^-3^ |  |  |  | *INMT-MINDY4* | 1.53 | 4.7 X 10^-3^ |
| *MRPL16* | 1.32 | 2.6 X 10^-3^ |  |  |  | *BRINP1* | 1.53 | 7.5 X 10^-3^ |
| *L3HYPDH* | 1.32 | 4.2 X 10^-3^ |  |  |  | *GRIN1* | 1.51 | 6.8 X 10^-3^ |
| *TMEM170B* | 1.31 | 2.3 X 10^-3^ |  |  |  | *SNX7* | 1.50 | 4.7 X 10^-3^ |
| *EDEM1* | 1.30 | 8.9 X 10^-3^ |  |  |  | *PLS1* | 1.50 | 6.8 X 10^-3^ |
| *TFIP11* | 1.29 | 7.9 X 10^-3^ |  |  |  | *ADAM9* | 1.50 | 4.2 X 10^-3^ |
| *LMO2* | 1.29 | 6.1 X 10^-3^ |  |  |  | *KIF26B* | 1.49 | 4.4 X 10^-3^ |
| *MRPS22* | 1.29 | 9.3 X 10^-3^ |  |  |  | *GPC4* | 1.49 | 6.8 X 10^-3^ |
| *GMPS* | 1.28 | 4.9 X 10^-3^ |  |  |  | *TACC1* | 1.49 | 1.9 X 10^-3^ |
| *ATP6V1A* | 1.27 | 1.2 X 10^-3^ |  |  |  | *ARHGEF35* | 1.49 | 4.7 X 10^-4^ |
| *PFDN2* | 1.27 | 5.4 X 10^-3^ |  |  |  | *STX3* | 1.48 | 2.8 X 10^-3^ |
| *PCGF5* | 1.27 | 5.1 X 10^-3^ |  |  |  | *HK1* | 1.48 | 5.4 X 10^-3^ |
| *RITA1* | 1.26 | 4.0 X 10^-3^ |  |  |  | *GCNT3* | 1.48 | 1.2 X 10^-3^ |
| *ARID1A* | 1.26 | 2.6 X 10^-3^ |  |  |  | *RBM17* | 1.48 | 2.8 X 10^-3^ |
| *PSMA6* | 1.25 | 7.2 X 10^-3^ |  |  |  | *MAK16* | 1.47 | 9.3 X 10^-3^ |
| *PSMA1* | 1.25 | 8.9 X 10^-3^ |  |  |  | *ADAMTS12* | 1.46 | 8.6 X 10^-3^ |
| *ADAL* | 1.25 | 2.1 X 10^-3^ |  |  |  | *ATF7-NPFF* | 1.46 | 3.0 X 10^-3^ |
| *ABCF2* | 1.23 | 2.3 X 10^-3^ |  |  |  | *SLC16A4* | 1.45 | 6.8 X 10^-3^ |
| *AKIRIN2* | 1.23 | 9.6 X 10^-3^ |  |  |  | *CFAP251* | 1.45 | 1.9 X 10^-3^ |
| *SRSF10* | 1.23 | 3.5 X 10^-3^ |  |  |  | *STK10* | 1.44 | 5.4 X 10^-3^ |
| *EXOSC8* | 1.23 | 5.8 X 10^-3^ |  |  |  | *FOXF2* | 1.43 | 8.2 X 10^-3^ |
| *USP12* | 1.22 | 1.6 X 10^-3^ |  |  |  | *GLB1L2* | 1.43 | 2.3 X 10^-3^ |
| *PSMD4* | 1.21 | 7.5 X 10^-3^ |  |  |  | *ANKRD26* | 1.43 | 2.3 X 10^-3^ |
| *METTL18* | 1.21 | 1.4 X 10^-3^ |  |  |  | *VPS41* | 1.42 | 2.8 X 10^-3^ |
| *RB1CC1* | 1.21 | 7.0 X 10^-4^ |  |  |  | *CTAGE8* | 1.42 | 6.8 X 10^-3^ |
| *SLC39A8* | 1.21 | 2.1 X 10^-3^ |  |  |  | *USP17L8* | 1.41 | 9.6 X 10^-3^ |
| *PSMA2* | 1.20 | 7.0 X 10^-3^ |  |  |  | *PHC1* | 1.41 | 1.9 X 10^-3^ |
| *TMEM129* | 1.19 | 2.6 X 10^-3^ |  |  |  | *CWC27* | 1.41 | 2.6 X 10^-3^ |
| *GOLGA8M* | 1.19 | 1.4 X 10^-3^ |  |  |  | *KMT2E* | 1.40 | 9.3 X 10^-4^ |
| *ABCA5* | 1.17 | 7.5 X 10^-3^ |  |  |  | *SET* | 1.40 | 1.9 X 10^-3^ |
| *RMDN2* | 1.17 | 2.6 X 10^-3^ |  |  |  | *ZKSCAN5* | 1.40 | 5.8 X 10^-3^ |
| *DCTN4* | 1.16 | 4.2 X 10^-3^ |  |  |  | *FIZ1* | 1.39 | 6.3 X 10^-3^ |
| *HIPK2* | 1.15 | 9.8 X 10^-3^ |  |  |  | *CENPK* | 1.39 | 9.8 X 10^-3^ |
| *CHCHD4* | 1.15 | 6.5 X 10^-3^ |  |  |  | *CNIH2* | 1.38 | 2.8 X 10^-3^ |
| *APOL6* | 1.14 | 6.1 X 10^-3^ |  |  |  | *SLK* | 1.37 | 5.4 X 10^-3^ |
| *ZBED9* | 1.12 | 2.3 X 10^-3^ |  |  |  | *IQCG* | 1.36 | 7.9 X 10^-3^ |
| *ABRAXAS2* | 1.12 | 3.0 X 10^-3^ |  |  |  | *KRIT1* | 1.35 | 7.2 X 10^-3^ |
| *ANKRD40* | 1.11 | 2.3 X 10^-3^ |  |  |  | *CFAP298* | 1.35 | 2.6 X 10^-3^ |
| *ZNF407* | 1.11 | 9.1 X 10^-3^ |  |  |  | *ZRANB2* | 1.35 | 2.8 X 10^-3^ |
| *NDUFA8* | 1.11 | 7.9 X 10^-3^ |  |  |  | *ALDOA* | 1.34 | 1.4 X 10^-3^ |
| *ME1* | 1.10 | 2.1 X 10^-3^ |  |  |  | *PCDHGA9* | 1.33 | 9.8 X 10^-3^ |
| *DCLRE1A* | 1.10 | 4.7 X 10^-4^ |  |  |  | *KIF2A* | 1.33 | 2.6 X 10^-3^ |
| *AOC2* | 1.07 | 6.5 X 10^-3^ |  |  |  | *JADE1* | 1.32 | 1.4 X 10^-3^ |
| *GFOD1* | 1.06 | 3.5 X 10^-3^ |  |  |  | *COG5* | 1.31 | 2.3 X 10^-4^ |
| *ZNF568* | 1.05 | 7.9 X 10^-3^ |  |  |  | *FGFR1OP2* | 1.31 | 8.6 X 10^-3^ |
| *ZNF138* | 1.03 | 2.1 X 10^-3^ |  |  |  | *STXBP3* | 1.30 | 1.2 X 10^-3^ |
| *GXYLT1* | 1.02 | 7.9 X 10^-3^ |  |  |  | *UBE2Z* | 1.30 | 7.7 X 10^-3^ |
| *SH3BGRL2* | 1.02 | 4.7 X 10^-3^ |  |  |  | *PLEKHA3* | 1.30 | 7.2 X 10^-3^ |
| *MPLKIP* | 1.01 | 1.6 X 10^-3^ |  |  |  | *WNT10A* | 1.29 | 7.5 X 10^-3^ |
| *SDE2* | 1.01 | 7.9 X 10^-3^ |  |  |  | *GLIPR1L1* | 1.28 | 3.7 X 10^-3^ |
|  |  |  |  |  |  | *TYRP1* | 1.28 | 2.6 X 10^-3^ |
|  |  |  |  |  |  | *SLC25A23* | 1.28 | 7.5 X 10^-3^ |
|  |  |  |  |  |  | *URGCP-MRPS24* | 1.27 | 2.6 X 10^-3^ |
|  |  |  |  |  |  | *BIN3* | 1.27 | 6.3 X 10^-3^ |
|  |  |  |  |  |  | *YTHDC1* | 1.25 | 8.9 X 10^-3^ |
|  |  |  |  |  |  | *MAP9* | 1.25 | 9.6 X 10^-3^ |
|  |  |  |  |  |  | *CUX1* | 1.24 | 8.6 X 10^-3^ |
|  |  |  |  |  |  | *NGRN* | 1.24 | 7.2 X 10^-3^ |
|  |  |  |  |  |  | *NCR3LG1* | 1.24 | 3.3 X 10^-3^ |
|  |  |  |  |  |  | *TSPAN3* | 1.23 | 6.1 X 10^-3^ |
|  |  |  |  |  |  | *PDPR* | 1.23 | 1.6 X 10^-3^ |
|  |  |  |  |  |  | *CBX3* | 1.23 | 3.7 X 10^-3^ |
|  |  |  |  |  |  | *RTCA* | 1.22 | 5.1 X 10^-3^ |
|  |  |  |  |  |  | *SUPT20H* | 1.22 | 6.1 X 10^-3^ |
|  |  |  |  |  |  | *CCZ1B* | 1.21 | 2.3 X 10^-4^ |
|  |  |  |  |  |  | *POMZP3* | 1.21 | 4.0 X 10^-3^ |
|  |  |  |  |  |  | *AFF1* | 1.21 | 4.2 X 10^-3^ |
|  |  |  |  |  |  | *KRT80* | 1.20 | 1.2 X 10^-3^ |
|  |  |  |  |  |  | *NAP1L1* | 1.20 | 7.0 X 10^-4^ |
|  |  |  |  |  |  | *PPP1R12A* | 1.19 | 3.5 X 10^-3^ |
|  |  |  |  |  |  | *BBS12* | 1.19 | 8.4 X 10^-3^ |
|  |  |  |  |  |  | *DNAJB6* | 1.19 | 9.3 X 10^-3^ |
|  |  |  |  |  |  | *MECOM* | 1.19 | 6.5 X 10^-3^ |
|  |  |  |  |  |  | *GNL3L* | 1.18 | 4.4 X 10^-3^ |
|  |  |  |  |  |  | *PMS2* | 1.17 | 2.8 X 10^-3^ |
|  |  |  |  |  |  | *LDHA* | 1.17 | 3.3 X 10^-3^ |
|  |  |  |  |  |  | *EIF4B* | 1.16 | 4.7 X 10^-4^ |
|  |  |  |  |  |  | *METRN* | 1.15 | 1.6 X 10^-3^ |
|  |  |  |  |  |  | *MMP10* | 1.14 | 4.7 X 10^-4^ |
|  |  |  |  |  |  | *LDOC1* | 1.13 | 8.9 X 10^-3^ |
|  |  |  |  |  |  | *RPL23* | 1.12 | 3.3 X 10^-3^ |
|  |  |  |  |  |  | *BTBD10* | 1.12 | 2.3 X 10^-3^ |
|  |  |  |  |  |  | *SELENOF* | 1.11 | 3.0 X 10^-3^ |
|  |  |  |  |  |  | *HNRNPDL* | 1.11 | 4.0 X 10^-3^ |
|  |  |  |  |  |  | *PCDHGA1* | 1.10 | 8.9 X 10^-3^ |
|  |  |  |  |  |  | *FAM83B* | 1.10 | 6.1 X 10^-3^ |
|  |  |  |  |  |  | *HNRNPA1* | 1.10 | 5.6 X 10^-3^ |
|  |  |  |  |  |  | *GNAL* | 1.09 | 7.2 X 10^-3^ |
|  |  |  |  |  |  | *GTF2F2* | 1.08 | 4.9 X 10^-3^ |
|  |  |  |  |  |  | *AKAP9* | 1.07 | 7.7 X 10^-3^ |
|  |  |  |  |  |  | *MOB1A* | 1.06 | 9.3 X 10^-4^ |
|  |  |  |  |  |  | *NOLC1* | 1.06 | 9.6 X 10^-3^ |
|  |  |  |  |  |  | *CAVIN3* | 1.06 | 8.4 X 10^-3^ |
|  |  |  |  |  |  | *PPP1R14B* | 1.05 | 4.9 X 10^-3^ |
|  |  |  |  |  |  | *ADAM17* | 1.04 | 8.2 X 10^-3^ |
|  |  |  |  |  |  | *SLC34A3* | 1.04 | 1.9 X 10^-3^ |
|  |  |  |  |  |  | *CIR1* | 1.04 | 1.4 X 10^-3^ |
|  |  |  |  |  |  | *FNDC11* | 1.03 | 8.4 X 10^-3^ |
|  |  |  |  |  |  | *NR2F2* | 1.03 | 8.6 X 10^-3^ |
|  |  |  |  |  |  | *DHX40* | 1.02 | 2.1 X 10^-3^ |
|  |  |  |  |  |  | *EPB41L2* | 1.02 | 2.8 X 10^-3^ |

HCC, hepatocellular carcinoma; int-CA, intermediate cell carcinoma; iCCA, intrahepatic cholangiocarcinoma

### Supplementary Table S5. Differentially altered CNAs (DCNAs) genes between HCC and iCCA.

| **cytoband** | **Fold difference**  **(HCC vs. iCCA)** | ***P*-value** | **cytoband** | **Fold difference**  **(HCC vs. iCCA)** | ***P*-value** |
| --- | --- | --- | --- | --- | --- |
| **DCNA for HCC**  (*n* = 58) | | | **DCNA for iCCA**  (*n* = 66) | | |
| chr1q21.3 | 0.30 | < 2.2 X 10^-16^ | chr4q21.1 | -0.21 | 6.2 X 10^-13^ |
| chr1q22 | 0.31 | < 2.2 X 10^-16^ | chr4q21.21 | -0.23 | 1.6 X 10^-14^ |
| chr1q23.1 | 0.30 | < 2.2 X 10^-16^ | chr4q21.22 | -0.22 | 6.2 X 10^-14^ |
| chr1q23.2 | 0.29 | < 2.2 X 10^-16^ | chr4q21.23 | -0.23 | 6.6 X 10^-15^ |
| chr1q23.3 | 0.30 | < 2.2 X 10^-16^ | chr4q21.3 | -0.22 | 1.5 X 10^-13^ |
| chr1q24.1 | 0.28 | < 2.2 X 10^-16^ | chr4q22.1 | -0.23 | 1.4 X 10^-14^ |
| chr1q24.2 | 0.28 | < 2.2 X 10^-16^ | chr4q22.2 | -0.23 | 1.6 X 10^-15^ |
| chr1q24.3 | 0.28 | < 2.2 X 10^-16^ | chr4q22.3 | -0.23 | 6.9 X 10^-16^ |
| chr1q25.1 | 0.29 | < 2.2 X 10^-16^ | chr4q23 | -0.23 | 5.3 X 10^-16^ |
| chr1q25.2 | 0.28 | < 2.2 X 10^-16^ | chr4q24 | -0.24 | 3.5 X 10^-16^ |
| chr1q25.3 | 0.27 | < 2.2 X 10^-16^ | chr4q25 | -0.21 | 2.4 X 10^-14^ |
| chr1q31.1 | 0.25 | 1.3 X 10^-14^ | chr4q28.3 | -0.20 | 9.8 X 10^-13^ |
| chr1q31.2 | 0.27 | < 2.2 X 10^-16^ | chr4q31.21 | -0.21 | 1.2 X 10^-13^ |
| chr1q31.3 | 0.28 | < 2.2 X 10^-16^ | chr4q31.22 | -0.21 | 7.1 X 10^-14^ |
| chr1q32.1 | 0.29 | < 2.2 X 10^-16^ | chr4q31.23 | -0.21 | 6.2 X 10^-14^ |
| chr1q32.2 | 0.30 | < 2.2 X 10^-16^ | chr4q31.3 | -0.21 | 1.8 X 10^-13^ |
| chr1q32.3 | 0.29 | < 2.2 X 10^-16^ | chr4q32.1 | -0.22 | 3.4 X 10^-14^ |
| chr1q41 | 0.26 | 1.6 X 10^-15^ | chr4q32.2 | -0.22 | 6.1 X 10^-14^ |
| chr1q42.11 | 0.25 | 1.9 X 10^-14^ | chr4q32.3 | -0.21 | 3.8 X 10^-13^ |
| chr1q42.12 | 0.26 | 8.9 X 10^-15^ | chr4q33 | -0.22 | 6.8 X 10^-13^ |
| chr1q42.13 | 0.26 | 8.9 X 10^-16^ | chr4q34.1 | -0.24 | 1.7 X 10^-14^ |
| chr1q42.2 | 0.28 | < 2.2 X 10^-16^ | chr4q34.2 | -0.23 | 3.1 X 10^-13^ |
| chr1q42.3 | 0.29 | < 2.2 X 10^-16^ | chr4q34.3 | -0.22 | 1.0 X 10^-11^ |
| chr1q43 | 0.28 | < 2.2 X 10^-16^ | chr4q35.1 | -0.20 | 1.6 X 10^-09^ |
| chr1q44 | 0.29 | < 2.2 X 10^-16^ | chr8p23.3 | -0.36 | 3.2 X 10^-15^ |
| chr6p25.3 | 0.21 | 4.6 X 10^-12^ | chr8p23.2 | -0.39 | 1.4 X 10^-17^ |
| chr6p25.2 | 0.22 | 5.3 X 10^-14^ | chr8p23.1 | -0.39 | 1.5 X 10^-17^ |
| chr6p25.1 | 0.22 | 8.0 X 10^-14^ | chr8p22 | -0.39 | 1.8 X 10^-18^ |
| chr6p24.3 | 0.23 | 8.0 X 10^-15^ | chr8p21.3 | -0.39 | 1.6 X 10^-18^ |
| chr6p24.2 | 0.22 | 3.4 X 10^-14^ | chr8p21.2 | -0.39 | 3.9 X 10^-19^ |
| chr6p24.1 | 0.21 | 2.2 X 10^-13^ | chr8p21.1 | -0.37 | 3.2 X 10^-17^ |
| chr6p23 | 0.21 | 3.5 X 10^-13^ | chr8p12 | -0.35 | 6.1 X 10^-17^ |
| chr6p22.3 | 0.20 | 1.9 X 10^-12^ | chr8p11.23 | -0.29 | 7.4 X 10^-11^ |
| chr8q12.1 | 0.23 | 2.1 X 10^-09^ | chr8p11.22 | -0.30 | 9.2 X 10^-12^ |
| chr8q12.2 | 0.24 | 3.9 X 10^-10^ | chr8p11.21 | -0.21 | 1.2 X 10^-07^ |
| chr8q12.3 | 0.25 | 3.0 X 10^-11^ | chr12p13.2 | -0.20 | 1.4 X 10^-14^ |
| chr8q13.1 | 0.27 | 1.1 X 10^-12^ | chr13q13.1 | -0.21 | 5.5 X 10^-12^ |
| chr8q13.2 | 0.29 | 5.1 X 10^-14^ | chr13q13.2 | -0.22 | 2.7 X 10^-12^ |
| chr8q13.3 | 0.31 | 2.0 X 10^-15^ | chr13q13.3 | -0.25 | 9.3 X 10^-15^ |
| chr8q21.11 | 0.29 | 3.6 X 10^-14^ | chr13q14.11 | -0.27 | 8.6 X 10^-17^ |
| chr8q21.12 | 0.28 | 3.1 X 10^-13^ | chr13q14.12 | -0.28 | 1.8 X 10^-16^ |
| chr8q21.13 | 0.28 | 2.8 X 10^-13^ | chr13q14.13 | -0.28 | 2.7 X 10^-17^ |
| chr8q21.2 | 0.29 | 7.6 X 10^-13^ | chr13q14.2 | -0.29 | 5.2 X 10^-17^ |
| chr8q21.3 | 0.28 | 8.3 X 10^-13^ | chr13q14.3 | -0.27 | 1.7 X 10^-16^ |
| chr8q22.1 | 0.30 | 6.6 X 10^-14^ | chr13q21.1 | -0.24 | 6.2 X 10^-14^ |
| chr8q22.2 | 0.30 | 4.0 X 10^-14^ | chr13q21.2 | -0.23 | 4.4 X 10^-13^ |
| chr8q22.3 | 0.30 | 9.3 X 10^-14^ | chr13q21.31 | -0.22 | 6.7 X 10^-12^ |
| chr8q23.1 | 0.30 | 2.3 X 10^-13^ | chr16q11.2 | -0.21 | 1.4 X 10^-11^ |
| chr8q23.2 | 0.29 | 1.6 X 10^-12^ | chr16q12.1 | -0.24 | 1.4 X 10^-16^ |
| chr8q23.3 | 0.29 | 2.4 X 10^-12^ | chr16q12.2 | -0.26 | 9.9 X 10^-18^ |
| chr8q24.11 | 0.28 | 4.9 X 10^-12^ | chr16q13 | -0.26 | 2.5 X 10^-17^ |
| chr8q24.12 | 0.29 | 1.3 X 10^-12^ | chr16q21 | -0.27 | 3.0 X 10^-18^ |
| chr8q24.13 | 0.28 | 6.4 X 10^-12^ | chr16q22.1 | -0.26 | 3.5 X 10^-17^ |
| chr8q24.21 | 0.26 | 3.9 X 10^-10^ | chr16q22.2 | -0.29 | 7.4 X 10^-20^ |
| chr8q24.22 | 0.28 | 6.4 X 10^-12^ | chr16q22.3 | -0.29 | 2.2 X 10^-20^ |
| chr8q24.23 | 0.28 | 6.2 X 10^-12^ | chr16q23.1 | -0.29 | 5.2 X 10^-20^ |
| chr8q24.3 | 0.28 | 3.0 X 10^-11^ | chr16q23.2 | -0.30 | 4.3 X 10^-20^ |
|  |  |  | chr16q23.3 | -0.31 | 9.8 X 10^-21^ |
|  |  |  | chr16q24.1 | -0.32 | 1.6 X 10^-21^ |
|  |  |  | chr16q24.2 | -0.32 | 4.5 X 10^-21^ |
|  |  |  | chr16q24.3 | -0.31 | 2.5 X 10^-20^ |
|  |  |  | chr17p13.3 | -0.32 | 2.3 X 10^-18^ |
|  |  |  | chr17p13.2 | -0.33 | 2.8 X 10^-20^ |
|  |  |  | chr17p13.1 | -0.35 | 1.5 X 10^-22^ |
|  |  |  | chr17p12 | -0.29 | 1.6 X 10^-16^ |

HCC, hepatocellular carcinoma; iCCA, intrahepatic cholangiocarcinom

**References**

[1] Ben-Porath I, Thomson MW, Carey VJ, Ge R, Bell GW, Regev A, et al. An embryonic stem cell-like gene expression signature in poorly differentiated aggressive human tumors. Nat Genet 2008;40:499-507.

[2] Woo HG, Lee JH, Yoon JH, Kim CY, Lee HS, Jang JJ, et al. Identification of a cholangiocarcinoma-like gene expression trait in hepatocellular carcinoma. Cancer Res 2010;70:3034-3041.

[3] Andrisani OM, Studach L, Merle P. Gene signatures in hepatocellular carcinoma (HCC). Semin Cancer Biol 2011;21:4-9.

[4] Liu YC, Yeh CT, Lin KH. Cancer Stem Cell Functions in Hepatocellular Carcinoma and Comprehensive Therapeutic Strategies. Cells 2020;9.

[5] Sun JH, Luo Q, Liu LL, Song GB. Liver cancer stem cell markers: Progression and therapeutic implications. World J Gastroenterol 2016;22:3547-3557.

[6] McGrath NA, Fu J, Gu SZ, Xie C. Targeting cancer stem cells in cholangiocarcinoma (Review). Int J Oncol 2020;57:397-408.

[7] Raggi C, Invernizzi P, Andersen JB. Impact of microenvironment and stem-like plasticity in cholangiocarcinoma: molecular networks and biological concepts. J Hepatol 2015;62:198-207.
